# IgSF21 promotes differentiation of inhibitory synapses via binding to neurexin2α

**DOI:** 10.1038/s41467-017-00333-w

**Published:** 2017-09-01

**Authors:** Yuko Tanabe, Yusuke Naito, Cristina Vasuta, Alfred Kihoon Lee, Youssouf Soumounou, Michael W. Linhoff, Hideto Takahashi

**Affiliations:** 1 Synapse Development and Plasticity Research Unit, Institut de RecherchesCliniques de Montréal, Montreal, QC Canada H2W 1R7; 2 Integrated Program in Neuroscience, McGill University, Montreal, QC Canada H3A 2B2; 3 Department of Anatomy and Neurobiology, Washington University School of Medicine, St. Louis, MO 63110 USA; 4 The Brain Research Centre and Department of Psychiatry, University of British Columbia, Vancouver, BC Canada V6T 2B5; 5 Vollum Institute, Oregon Health and Science University, Portland, OR 97239 USA; 6 Department of Medicine, Université de Montréal, Montreal, QC Canada H3T 1J4; 7 Division of Experimental Medicine, McGill University, Montreal, QC Canada H3A 0G4

## Abstract

Coordinated development of excitatory and inhibitory synapses is essential for higher brain function, and impairment in this development is associated with neuropsychiatric disorders. In contrast to the large body of accumulated evidence regarding excitatory synapse development, little is known about synaptic adhesion and organization mechanisms underlying inhibitory synapse development. Through unbiased expression screens and proteomics, we identified immunoglobulin superfamily member 21 (IgSF21) as a neurexin2α-interacting membrane protein that selectively induces inhibitory presynaptic differentiation. IgSF21 localizes postsynaptically and recruits axonal neurexin2α in a trans-interaction manner. Deleting IgSF21 in mice impairs inhibitory presynaptic organization, especially in the hippocampal CA1 stratum radiatum, and also diminishes GABA-mediated synaptic transmission in hippocampal CA1 neurons without affecting their excitatory synapses. Finally, mice lacking IgSF21 show a sensorimotor gating deficit. These findings suggest that IgSF21 selectively regulates inhibitory presynaptic differentiation through interacting with presynaptic neurexin2α and plays a crucial role in synaptic inhibition in the brain.

## Abstract (web summary)

Molecular mechanisms regulating the development of inhibitory synapses are poorly understood. Here the authors show that IgSF21 interacts with neurexin2α to induce presynaptic differentiation of inhibitory synapses, and that mice lacking IgSF21 exhibit deficits in inhibitory synaptic transmission.

## Introduction

γ-Aminobutyric acid (GABA)-mediated synaptic transmission determines neuronal excitability and the firing patterns of target neurons^1^ and also regulates neuronal circuit formation^2–5^. In the mature brain, each neuron receives excitatory and inhibitory synaptic inputs from glutamatergic neurons and GABAergic interneurons, respectively, and a proper balance between these inputs is needed to maintain normal brain functions^6, 7^. To fine-tune neuronal excitability, GABAergic innervation is complex with different types of interneurons forming inhibitory synapses in a cell type-specific and compartment-specific manner^8–11^. The mechanisms underlying the development of these complex GABAergic innervation patterns are poorly understood. Recent evidence indicates that impaired development of central inhibitory synapses is fundamental to the etiology of neuropsychiatric disorders such as schizophrenia^12, 13^, autism spectrum disorders (ASD)^7, 14^ and anxiety^15^. Thus, the importance of expanding our understanding of the molecular mechanisms of GABAergic synapse development has been highlighted in recent years.

Synapse development requires not only physical contact between axons and target neurons but also chemically matched presynaptic and postsynaptic differentiation^16, 17^. Synapse organizing complexes, trans-synaptic adhesion complexes with the ability to induce synaptic differentiation, act as essential molecular signals for synapse development^18–21^. The neuroligin (NLG)–neurexin (NRX) complex is the best-studied synapse organizing complex^19, 22^, but there are many others including LRRTMs-NRXs^23, 24^, GluRδ-cerebellin-NRXs^25, 26^, TrkC-PTPσ^27, 28^, Slitrks-PTPδ/σ^29, 30^ and NGL3-LAR^31^. Notably, only Slitrk3-containing and NLG2/4-containing complexes have been reported to function selectively or preferentially in inhibitory synapse development^29, 30, 32–34^.

The relatively sparse identification of synapse organizing complexes selectively regulating inhibitory synapse development could be due in part to the complex nature of GABAergic interneuron innervation^8–11^. Neuronal circuits contain many types of GABAergic interneurons, and distinct subtypes innervate specific subcellular compartments of target neurons (the dendrites, the soma, or the axon initial segment), consequently providing multifaceted inhibitory impacts^8, 11^. The heterogeneous nature of GABAergic synaptic connections suggests that there must be many types of adhesion complexes regulating inhibitory synapses.

To identify novel synapse organizing proteins, an artificial synapse formation assay using fibroblast-neuron co-cultures to visualize the synapse-inducing (“synaptogenic”) activity of molecules of interest was utilized^18, 23, 27, 35^. Combining this assay with a rat brain cDNA library in an unbiased screen isolated a few novel synapse organizers including LRRTM1^23^, TrkC^27^, and calsyntenin-3^35^. Here, we report the additional isolation of immunoglobulin superfamily member 21 (IgSF21) as a novel synaptogenic membrane protein that induces inhibitory, but not excitatory, presynaptic differentiation. Through another unbiased proteomics screen, we isolated NRX2α as an IgSF21-interacting presynaptic organizer. IgSF21 recruits NRX2α to presynaptic sites in a trans-interaction manner and this interaction is essential for the synaptogenic activity of IgSF21. To date, there is no published study characterizing the physiological roles of IgSF21. Thus, to investigate its function in the central nervous system, we generated and comprehensively characterized IgSF21 mutant mice. We found that IgSF21 positively controls inhibitory presynaptic organization and GABA-mediated synaptic transmission in the hippocampal CA1 pyramidal neurons and that IgSF21 is required for normal sensorimotor gating. Together, our results suggest that IgSF21 selectively organizes inhibitory synapses through its trans-synaptic interaction with axonal NRX2α and that this is important for normal brain function.

## Results

### Inhibitory presynaptic differentiation induced by IgSF21

**Fig. 1:**
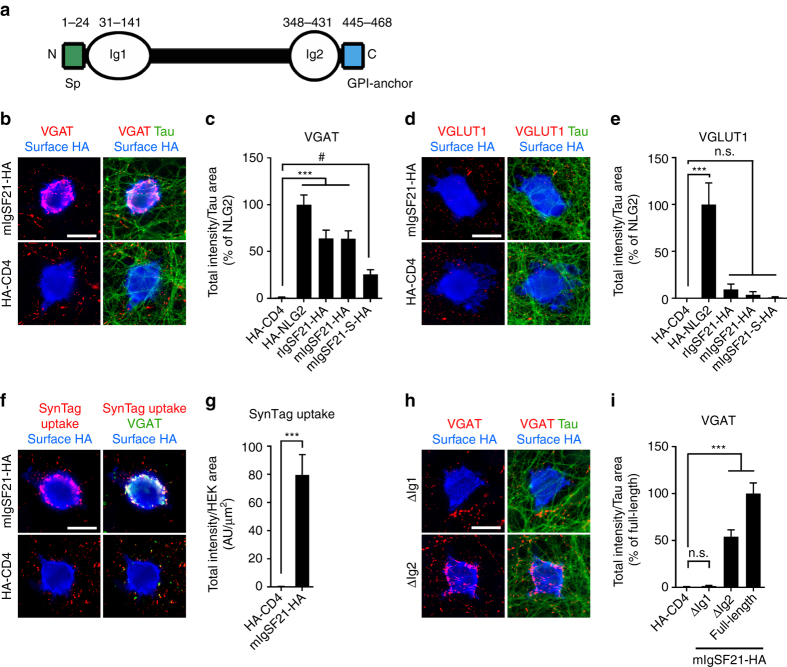
IgSF21 induces functional inhibitory presynaptic differentiation in coculture assays. **a** Domain structure of IgSF21. IgSF21 has two immunoglobulin-like domains (Ig1 and Ig2), an N-terminal signal peptide (Sp) and a C-terminal signal peptide for glycosylphosphatidylinositol (GPI)-anchor modification (GPI-anchor). **b**, **d** HEK293 cells expressing mouse IgSF21 tagged with HA (mIgSF21-HA) induce clustering of VGAT **b**, but not that of VGLUT1 **d**, along contacting axons (labeled with dephosphorylated tau) in hippocampal neuron cultures. HEK293 cells expressing HA-CD4 as a negative control do not induce clustering of either VGAT or VGLUT1. **c**, **e** Quantification of the total intensity of VGAT **c** and VGLUT1 **e** puncta associated with HEK293 cells expressing the indicated HA-tagged proteins and not associated with MAP2 divided by tau-positive axon-contact area, expressed as a percentage of the value for the HA-neuroligin2 (HA-NLG2) positive control. *n* ≥ 20 cells for each construct from three independent experiments, Kruskal–Wallis one-way ANOVA, *P* < 0.0001 for VGAT and VGLUT1. ^#^ *P = *0.0112 and ***P < 0.0001 compared with HA-CD4 by Dunn’s multiple comparisons test. **f**, **g** HEK293 cells expressing mIgSF21-HA induce presynaptic terminals with active synaptic vesicle recycling, as assessed by incubating live neurons with antibodies recognizing the synaptotagmin I (SynTag) luminal domain. mIgSF21-HA, but not HA-CD4, induces SynTag uptake (red) associated with VGAT accumulation (green). *n* > 25 cells for each construct from three independent experiments, Mann–Whitney test, ***P < 0.0001. **h**, **i** HEK293 cells expressing mIgSF21 lacking the Ig1 domain (∆Ig1) do not induce VGAT clustering. *n* ≥ 20 cells for each construct from three independent experiments, Kruskal–Wallis one-way ANOVA, *P* < 0.0001. ***P < 0.0001 compared with HA-CD4 by Dunn’s multiple comparisons test. n.s., not significant. *Scale bars* represent 20 μm **b**, **d**, **f**, **h**. Data are presented as mean ± s.e.m

To identify novel synaptogenic molecules, we performed an unbiased expression screen based on an artificial synapse formation assay in which hippocampal neurons are cocultured with fibroblasts transfected with rat brain cDNA library pools^23, 27, 35^. From the positive pool PB243, we isolated IgSF21 as a synaptogenic clone, as assessed by the accumulation of the presynaptic marker synapsin I in the coculture assay (Supplementary Fig. 1a, b). IgSF21 has two extracellular immunoglobulin-like domains (Ig1 and Ig2) and a putative C-terminal signal sequence for a glycosylphosphatidylinositol (GPI)-anchor modification but no transmembrane domain (Fig. 1a and Supplementary Fig. 1c, d). IgSF21 is expressed on the cell surface but can be stripped from the surface using phosphatidylinositol-specific phospholipase C (PI-PLC), confirming that IgSF21 is a GPI-anchored protein (Supplementary Fig. 1e, f). *Igsf21* is a vertebrate-specific gene whose Ig domains are highly conserved between species (Supplementary Fig. 1c, d). Mice express at least two IgSF21 isoforms; one is a homolog (mIgSF21) of rat IgSF21 (rIgSF21) with a longer linker between the two Ig domains and the other is an alternatively spliced isoform with a shorter linker (mIgSF21-S). Thus, we next tested whether each IgSF21 isoform induces excitatory and/or inhibitory presynaptic differentiation in coculture assays using HEK293 cells expressing HA-tagged IgSF21 proteins. Notably, rIgSF21, mIgSF21 and mIgSF21-S all induced the accumulation of VGAT, an inhibitory (GABAergic) presynaptic marker, but not that of VGLUT1, an excitatory (glutamatergic) presynaptic marker, at contacting axons of hippocampal neurons (Fig. 1b–e) or cortical neurons (Supplementary Fig. 2a–d), indicating that IgSF21 selectively induces inhibitory presynaptic differentiation. For the remainder of this study, we focused on the IgSF21 isoform with a longer linker because the synaptogenic activity of mIgSF21-S was very weak (Fig. 1c) although surface expression of mIgSF21-S is comparable to that of mIgSF21 (Supplementary Fig. 3a). IgSF21 also induced the uptake of an antibody directed against the synaptotagmin I luminal domain (SynTag; Fig. 1f, g), which occurs only during active recycling of synaptic vesicles (SVs), suggesting that IgSF21 induces functional presynaptic differentiation. On the other hand, IgSF21 did not induce the accumulation of either gephyrin or PSD-95 in contacting dendrites (Supplementary Fig. 2e–h), showing that IgSF21 has no postsynaptic induction activity. We further determined the domain responsible for IgSF21 synaptogenic activity using deletion constructs in the coculture assay. IgSF21 lacking Ig1 did not induce VGAT accumulation whereas IgSF21 lacking Ig2 did but had only about half the synaptogenic activity of full-length IgSF21 (Fig. 1h, i). There was no difference in surface expression of full-length IgSF21, IgSF21 lacking Ig1 or IgSF21 lacking Ig2 (Supplementary Fig. 3b), indicating that the differences in their synaptogenic activity are not due to differing surface expression levels. These results demonstrate that the Ig1 domain of IgSF21 is indispensable for synaptogenic activity whereas its Ig2 domain is secondarily responsible for the activity. Thus, IgSF21 selectively induces the differentiation of functional inhibitory presynaptic terminals primarily through its Ig1 domain.

### IgSF21 expression and localization

**Fig. 2:**
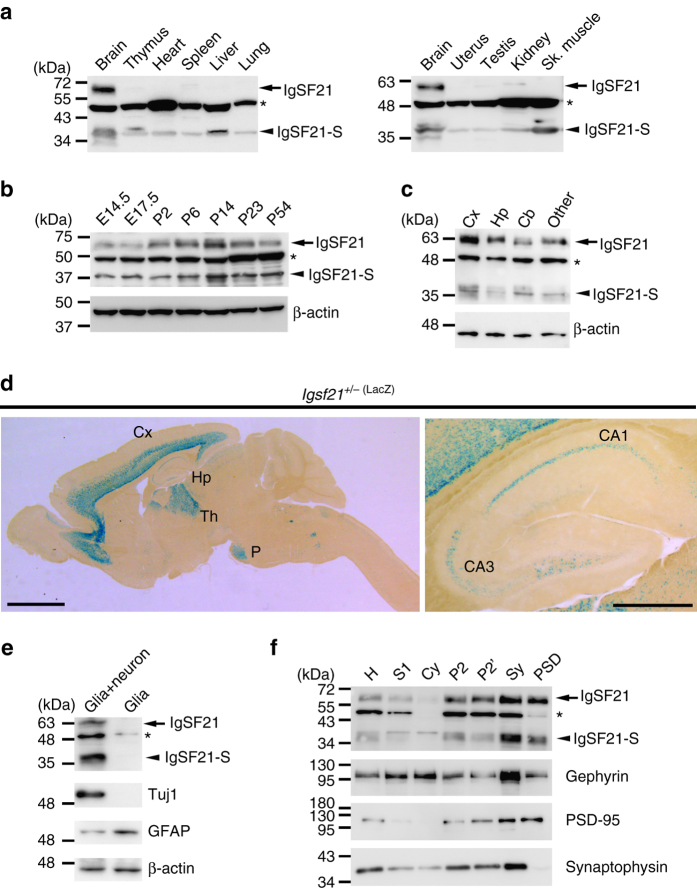
The long isoform of IgSF21 is preferentially expressed in the brain and localized at postsynaptic sites in neurons. **a** Expression of IgSF21 protein in mouse tissue at two postnatal weeks. Western blots show the preferential expression of the IgSF21 long isoform in the brain. Sk. muscle, skeletal muscle. **b** Expression of IgSF21 protein during mouse brain development. IgSF21 is highly expressed at 2–3 postnatal weeks and remains expressed in the adult. E, embryonic; P, postnatal. **c** Expression of IgSF21 protein in several mouse brain regions at four postnatal weeks. Cx, cortex; Hp, hippocampus; Cb, cerebellum; Other, other brain regions. **d** Images of X-gal staining of *Igsf21* ^*+/−(LacZ)*^ mouse brain at four postnatal weeks show that the *Igsf21* gene is preferentially expressed in the deep layers of the cortex (Cx), the pyramidal cell layer of the CA1 and CA3 regions of the hippocampus (Hp; *left image*, CA1, CA3; *right image*), the thalamus (Th), and the pons (P). **e** Immunoblot of cell lysates from relatively pure primary glial cultures and mixed cultures of glial cells and neurons. IgSF21 is expressed in neurons but not glial cells. Tuj1, a neuron-specific class III β-tubulin; GFAP, glial fibrillary acid protein. **f** Expression of IgSF21 protein in mouse brain fractions at three postnatal weeks. IgSF21 is enriched in the crude synaptosome (P2 and P2’), synaptosome (Sy) and postsynaptic density (PSD) fractions. H, homogenate; S1, supernatant 1; Cy, cytoplasm. *Scale bars* represent 2 mm (**d**, *left*) and 500 μm (**d**, *right*). *Arrow*, IgSF21 (~60 kDa); * non-specific bands. Uncropped blots for **a**, **b**, **c**, **e** and **f** are shown in Supplementary Fig. 10

We next examined the expression pattern of IgSF21 in mouse tissues. Immunoblot analysis revealed that the long form of IgSF21 (~60 kDa) is predominantly expressed in the brain, whereas the IgSF21 short isoform (~35 kDa) is also expressed in other organs (Fig. 2a). IgSF21 is expressed in the brain at both embryonic and postnatal stages including the adult stage (Fig. 2b). In particular, the period of highest expression of the long isoform is at around two postnatal weeks, coinciding with the peak period of synaptogenesis. IgSF21 displays fairly broad expression in the brain: it is expressed in the cortex and the hippocampus as well as other brain regions (Fig. 2c). To more precisely assess *Igsf21* gene expression, we generated mice in which a gene trap cassette containing a *LacZ* reporter gene with a splicing acceptor was inserted into the *Igsf21* locus^36^ (Supplementary Fig. 4a–c). In mice heterozygous for this allele (*Igsf21* ^*+/−(LacZ)*^), β-galactosidase encoded by the *LacZ* gene is expressed under the control of the *Igsf21* promoter, allowing us to analyze *Igsf21* expression by examining β-gal enzymatic activity using X-gal as a substrate. The X-gal staining of brain sections from these mice demonstrated that at four postnatal weeks, the *Igsf21* gene is highly expressed in the pyramidal cell layer of the dorsal and ventral hippocampal CA1 and CA3 regions (Fig. 2d and Supplementary Fig. 5a), layers 5 and 6 of the cortex (Fig. 2d and Supplementary Fig. 5b), the thalamus and the pons (Fig. 2d) and weakly expressed in the cerebellum (Fig. 2d and Supplementary Fig. 5c).

At the cellular level, IgSF21 is expressed in neurons but not in glia (Fig. 2e). Upon fractionation of mouse brains, IgSF21 protein is present in the synaptosome and postsynaptic density (PSD) fractions (Fig. 2f), indicating that IgSF21 is localized postsynaptically in neurons. We further investigated the subcellular localization of IgSF21 by expressing a construct consisting of IgSF21 tagged with HA (IgSF21-HA) in hippocampal neurons. IgSF21-HA predominantly targeted to dendrites but also to axons (Supplementary Fig. 6a, b). IgSF21-HA also exhibited a diffuse distribution with a punctate pattern in dendrites. Some IgSF21-HA puncta on dendrites were juxtaposed to and/or overlapped with a subset of VGAT puncta. IgSF21-HA puncta on dendrites were associated significantly more with VGAT puncta than with VGLUT1 puncta (Supplementary Fig. 6c, d). Together these data suggest that IgSF21 accumulates at postsynaptic sites of a subset of inhibitory synapses.

### Selective interaction of IgSF21 with Neurexin2α

**Fig. 3:**
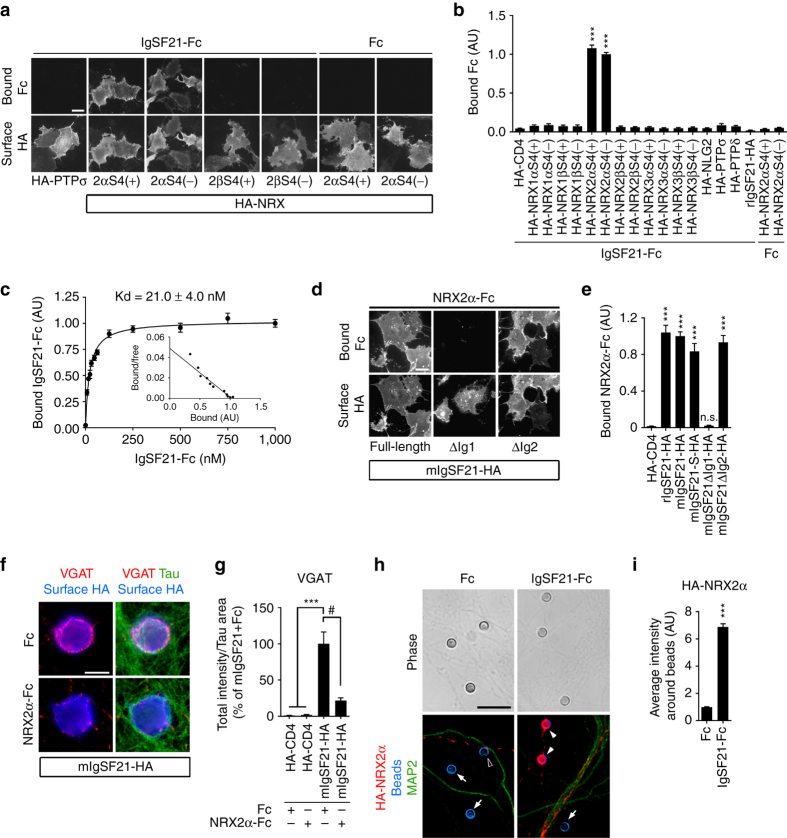
IgSF21 binds and recruits neurexin2α for inhibitory presynaptic differentiation. **a**, **b** IgSF21-Fc binds specifically to COS-7 cells expressing extracellularly HA-tagged neurexin2α (HA-NRX2α) regardless of alternative splicing at site 4 (S4). For the binding assays, HA-NRX1αS1(+) S2(+) S3(+) S4(+/−), HA-NRX2αS1(+) S2(+) S3(+) S4(+/−) and HA-NRX3αS1(−) S2(−) S3(+) S4(+/−) variants were used. *n* = 30 cells for each construct from three independent experiments, Kruskal–Wallis one-way ANOVA, *P* < 0.0001. ***P < 0.0001 compared with HA-CD4 by Dunn’s multiple comparisons test. **c** Scatchard plot analysis shows that the Kd for the binding of IgSF21-Fc to HA-NRX2αS4(-) is 21.0 nM (*n* = 30 cells). **d**, **e** NRX2α-Fc binds to rat and mouse full-length IgSF21-HA (rIgSF21-HA, mIgSF21-HA), the mIgSF21-short isoform (mIgSF21S)-HA and mIgSF21-HA lacking Ig2 (∆Ig2), but not to mIgSF21-HA lacking Ig1 (∆Ig1). *n* = 30 cells for each construct from three independent experiments, Kruskal–Wallis one-way ANOVA, *P* < 0.0001. ***P < 0.0001 compared with HA-CD4 by Dunn’s multiple comparisons test. n.s., not significant. **f**, **g** Soluble NRX2α-Fc (15 nM) added to the coculture medium suppresses VGAT accumulation induced by mIgSF21-HA. *n* > 30 cells for each condition from three independent experiments, Kruskal–Wallis one-way ANOVA, *P* < 0.0001. ^#^ *P* = 0.0105 and ***P < 0.0001 by Dunn’s multiple comparisons test. **h**, **i** IgSF21-Fc-coated beads (filled arrowheads) induce NRX2α accumulation in contacting axons of cultured hippocampal neurons transfected with HA-NRX2α. An open arrowhead indicates an Fc-coated bead that physically contacts a transfected axon but has no significant accumulation of HA-NRX2α. *Arrows* indicate beads not contacting transfected axons. *n* = 75 beads for each condition from three independent experiments, Mann–Whitney test, ***P < 0.0001. *Scale bars* represent 30 μm **a**, **d** and 10 μm **f**, **h**. Data are presented as mean ± s.e.m

To identify IgSF21-interacting presynaptic organizers, we performed an unbiased proteomics screen using protein-A sepharose magnetic beads coated with recombinant IgSF21 fused to the human immunoglobulin Fc region (IgSF21-Fc) (Supplementary Fig. 7). We applied IgSF21-Fc-coated magnetic beads onto hippocampal neuron cultures. First, we observed that IgSF21-Fc-coated beads induced the accumulation of VGAT but not VGLUT1 (Supplementary Fig. 7a–c), confirming that the IgSF21-Fc recombinant protein retains selective inhibitory presynaptic differentiation activity. We then performed protein crosslinkage of surface-bound proteins to IgSF21-Fc using non-permeable 3,3ʹ-dithiobis(sulfosuccinimidyl propionate) (DTSSP). Our proteomics analysis identified NRX2α and PTPσ as membrane proteins pulled down by IgSF21-Fc-coated beads (Supplementary Fig. 7d–f). In a cell surface binding assay, we determined that IgSF21-Fc interacts with NRX2α but not PTPσ (Fig. 3a, b).

We next determined which of the many NRX family isoforms^37^ interact with IgSF21. Interestingly, IgSF21-Fc interacted only with NRX2α and not with any other NRX isoforms (Fig. 3a, b). The binding of IgSF21-Fc with NRX2α is at high affinity (Kd = 21.0 nM) (Fig. 3c) and is independent of extracellular calcium and is unlikely to require inserts at any of the NRX2α-splicing sites, S1-S4 (Supplementary Fig. 8a–c). We further determined the domain of NRX2α that is responsible for IgSF21 binding by deletion analysis. The first LNS (laminin, neurexin and sex hormone-binding globulin-like) domain (LNS1) of NRX2α was necessary and sufficient for IgSF21-binding (Supplementary Fig. 8d, e). Conversely, IgSF21 Ig1 was necessary for NRX2α-binding and IgSF21 Ig2 was dispensable for NRX2α-binding (Fig. 3d, e). Thus, the Ig1 domain of IgSF21 is primarily responsible for both IgSF21 synaptogenic activity (Fig. 1h, i) and NRX2α interaction (Fig. 3d, e), suggesting that NRX2α acts as a functional IgSF21 receptor. Further, in an artificial synapse formation assay, VGAT accumulation induced by mIgSF21-HA was significantly inhibited by the addition of soluble NRX2α-Fc to the coculture medium (Fig. 3f, g). This confirms that IgSF21-NRX2α interaction is involved in the synaptogenic activity of IgSF21. Next, we tested whether IgSF21 can recruit axonal NRX2α by adding IgSF21-Fc-coated beads onto hippocampal neurons expressing HA-tagged NRX2α (HA-NRX2α). IgSF21-Fc-coated beads induced strong accumulation of HA-NRX2α in contacting transfected axons, whereas Fc-coated beads (a negative control) did not (Fig. 3h, i). Together, these data indicate that IgSF21 binds and recruits axonal NRX2α in a trans-interaction manner and that this interaction is essential for the synaptogenic activity of IgSF21.

### Impaired inhibitory synaptic organization in *Igsf21*^−/−^ mice

**Fig. 4:**
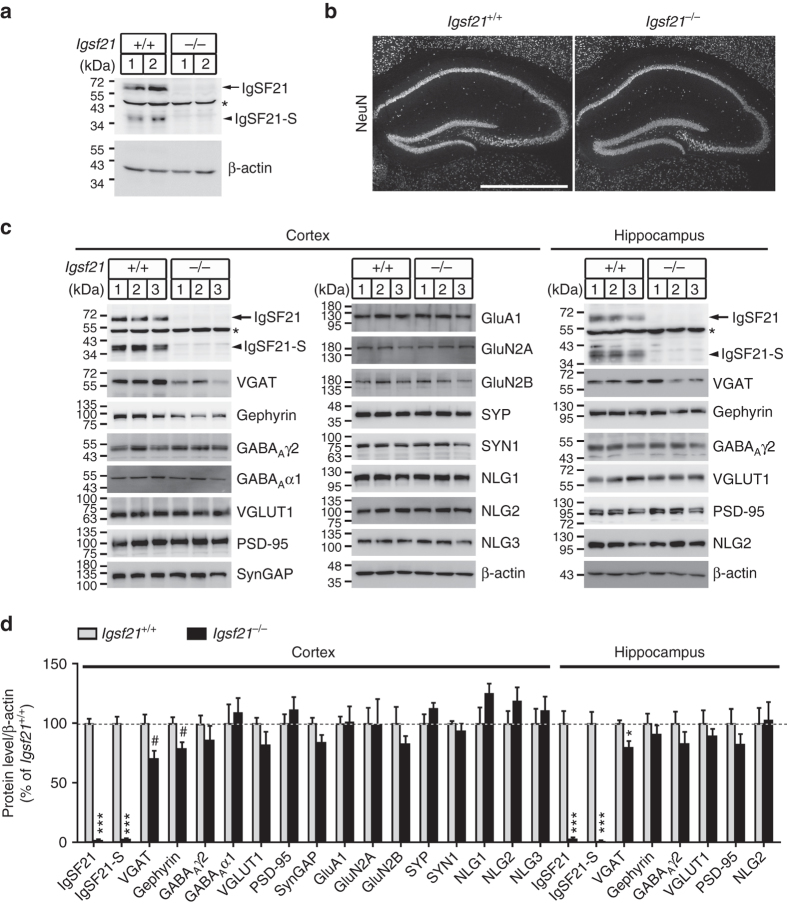
Inhibitory synaptic protein levels are reduced in synaptosomes from *Igsf21* ^−/−^ mice. **a** An immunoblot shows loss of IgSF21 and IgSF21-S protein expression in total brain lysate from *Igsf21* ^−/−^ mice. The labels 1 and 2 indicate samples from different mice. **b** Confocal NeuN images of the hippocampus of *Igsf21* ^+/+^ and *Igsf21* ^−/−^mice at four postnatal weeks show that the gross morphology is normal. The *scale bar* represents 1 mm. **c** Representative immunoblots of the indicated proteins in synaptosomes from the cerebral cortex and from the hippocampus of *Igsf21* ^+/+^ and *Igsf21* ^−/−^ mice. The labels 1, 2 and 3 indicate samples from different mice. **d** Quantification of synaptic protein expression normalized to β-actin loading controls in synaptosomes from the cortex and the hippocampus, expressed as a percentage of the values in *Igsf21* ^+/+^ mice. *n* = 6 samples per protein per genotype for cortex, with each *n* representing a cortex from one mouse. *n* = 7 samples per protein per genotype for hippocampus, with each *n* representing pooled hippocampi from four mice. Unpaired *t*-tests, ^#^ *P < *0.05 (Cortex: VGAT, *P* = 0.0124; gephyrin, *P* = 0.0152), *P < 0.01 (Hippocampus: VGAT, *P* = 0.0044), ***P < 0.0001. *Arrow*, IgSF21 (~60 kDa); *arrowhead*, IgSF21-S (~35 kDa); *non-specific bands. SYP, synaptophysin; SYN1, synapsin I; NLG1/2/3, neuroligin1/2/3. Data are presented as mean ± s.e.m. Uncropped blots for **a** and **c** are shown in Supplementary Figs. 10 and 11, respectively

To investigate the *in vivo* roles of endogenous IgSF21 in synapse organization, we analyzed IgSF21 homozygous mutant mice (*Igsf21* ^−/−^) generated by a gene trapping strategy^36^ (Supplementary Fig. 4a–c). We confirmed complete loss of mIgSF21 and mIgSF21-S protein expression in the *Igsf21* ^−/−^ mouse brain by immunoblotting (Fig. 4a) and loss of mRNAs encoding mIgSF21, mIgSF21-S and any other possible IgSF21 isoforms possessing Ig1 by RT-PCR (Supplementary Fig. 4d, e).

*Igsf21* ^−/−^mice grew normally (Supplementary Fig. 4f) and bore no gross brain abnormalities including in the hippocampus (Fig. 4b). We next checked the levels of synaptic proteins in the synaptosomal fraction of the cerebral cortex. Immunoblot analysis showed that synaptosomes from *Igsf21* ^−/−^mice contained less VGAT and gephyrin than those from wild-type (*Igsf21* ^+/+^) mice but there were no changes in the levels of other synaptic proteins including GABA_A_ receptors and excitatory synaptic proteins such as VGLUT1, PSD-95 and glutamate receptors (Fig. 4c, d). There were no changes in the NLG1, 2 and 3 levels (Fig. 4c, d), indicating that the reduction of VGAT and gephyrin in the *Igsf21* ^−/−^ synaptosome fraction cannot be due to a reduction in NLG1/2/3 expression. Thus, deleting IgSF21 causes selective deficits in synaptosomal inhibitory synaptic components. A similar reduction of VGAT, but not gephyrin, GABA_A_ receptor γ2 subunit, VGLUT1, PSD-95 or NLG2, occurs in synaptosomes from the hippocampus of *Igsf21* ^−/−^mice (Fig. 4c, d).

We next performed immunohistochemistry on coronal brain sections from *Igsf21* ^−/−^mice. Given that the *Igsf21* gene is highly expressed in layers 5 and 6 of the cerebral cortex (Supplementary Fig. 5b), we first analyzed immunofluorescence for inhibitory synaptic markers in layer 5 of the somatosensory cortex. Consistent with the immunoblot analysis (Fig. 4c, d), *Igsf21* ^−/−^ mice had a significant decrease in immunoreactivity for VGAT and gephyrin in layer 5 of the somatosensory cortex (Supplementary Fig. 9a, b).

**Fig. 5:**
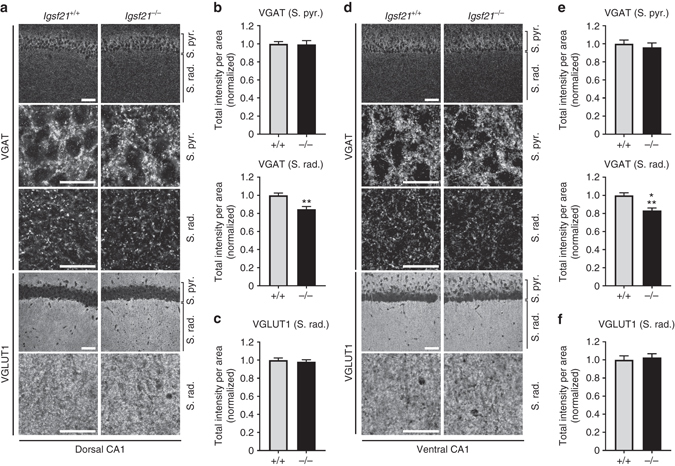
VGAT immunofluorescence is reduced in the hippocampal CA1 stratum radiatum in *Igsf21* ^−/−^ mice. **a**, **d** Double immunolabeling for VGAT and VGLUT1 in the dorsal **a** and ventral **d** hippocampal CA1 region of *Igsf21* ^+/+^ and *Igsf21* ^−/−^ mice. The VGAT signal in the stratum radiatum (S. rad.), but not the stratum pyramidale (S. pyr.), is slightly lower in *Igsf21* ^−/−^ mice compared to that in *Igsf21* ^+/+^mice in both the dorsal and the ventral CA1 regions. The VGLUT1 signal in *Igsf21* ^−/−^ mice is comparable to that in *Igsf21* ^+/+^mice. **b**, **c**, **e**, **f** Quantification of the total intensity of VGAT puncta in the stratum pyramidale and the stratum radiatum in the dorsal CA1 **b** and ventral CA1 **e**, and VGLUT1 puncta in the stratum radiatum in the dorsal CA1 **c** and ventral CA1 **f**. VGAT and VGLUT1 intensity in the stratum radiatum was measured in the same fields. Intensity values are normalized to the mean value of *Igsf21* ^+/+^ control. Dorsal CA1, S. pyr.: *n* = 30 fields from 4 *Igsf21* ^+/+^ mice and 32 fields from 4 *Igsf21* ^−/−^ mice. S. rad.: *n* = 90 and 96 fields from 4 *Igsf21* ^+/+^ mice and 4 *Igsf21* ^−/−^ mice, respectively. Ventral CA1, S. pyr.: *n* = 36 fields from 4 *Igsf21* ^+/+^ mice and 36 fields from 4 *Igsf21* ^−/−^ mice. S. rad.: *n* = 90 fields from 4 *Igsf21* ^+/+^ mice and 90 fields from 4 *Igsf21* ^−/−^ mice. Mann–Whitney test, **P = 0.0005 **b**, ***P < 0.0001 **e**. *Scale bars* represent 50 μm (**a**, **d**, low magnification images) and 20 μm (**a**, **d**, high magnification images). S. pyr., stratum pyramidale; S. rad., stratum radiatum. Data are presented as mean ± s.e.m

To further assess synaptic deficits in the *Igsf21* ^−/−^ mice, we focused on the CA1 hippocampus because the *Igsf21* gene is significantly expressed in the CA1 region and because this region allows for easy segregation of the somatic and dendritic regions of pyramidal neurons. In double immunostaining analysis of the CA1 stratum radiatum, which contains mainly dendritic synapses, the total intensity of VGAT puncta was 20% lower in *Igsf21* ^−/−^mice than in *Igsf21* ^+/+^ littermates in the dorsal hippocampus (Fig. 5a, b) and in the ventral hippocampus (Fig. 5d, e), while there was no difference in total VGLUT1 intensity (Fig. 5a, c, d, f). Notably, no change in VGAT expression was detected in the stratum pyramidale (Fig. 5a, b, d, e), which contains mainly perisomatic synapses. These data therefore suggest that *Igsf21* ^−/−^ mice may display impaired formation of inhibitory synapses and/or impaired organization of inhibitory presynaptic terminals, especially in the CA1 stratum radiatum of both the dorsal and the ventral hippocampus.

**Fig. 6:**
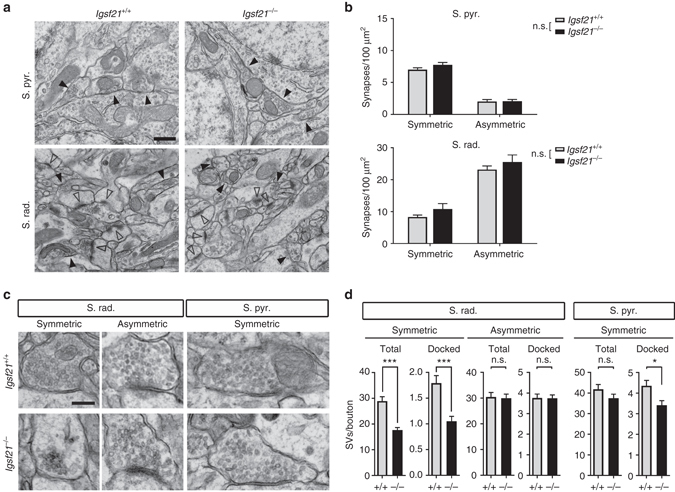
Symmetric synapses on dendrites in the hippocampal CA1 stratum radiatum of *Igsf21* ^−/−^ mice have fewer presynaptic vesicles. **a** Electron micrographs showing the hippocampal CA1 stratum pyramidale (S. pyr.) and stratum radiatum (S. rad.) of *Igsf21* ^+/+^ and *Igsf21* ^−/−^ mice at four postnatal weeks. Filled and open arrowheads indicate symmetric and asymmetric synapses, respectively. **b** Quantification of the number of symmetric and asymmetric synapses in the stratum pyramidale and the stratum radiatum. *n* = 6 mice for each genotype, with data averaged from 30 fields (6 and 10 combined images/field for S. pyr. and S. rad., respectively) per mouse. Two-way ANOVA, *F* (1, 20) = 1.798, *P* = 0.1949 between genotypes for S. pyr. Two-way ANOVA, *F* (1, 20) = 2.694, *P* = 0.1164 between genotypes for S. rad. n.s., not significant. **c** Electron micrographs showing symmetric and asymmetric synapses on dendritic regions in the stratum radiatum and symmetric synapses on perisomatic regions in the stratum pyramidale of *Igsf21* ^+/+^ and *Igsf21* ^−/−^ mice. **d** Quantification of the number of total and docked synaptic vesicles (SVs) per bouton of symmetric and asymmetric synapses on dendritic regions in the stratum radiatum and of symmetric synapses on perisomatic regions in the stratum pyramidale. *n* > 110 synapses for each type of synapse from 6 mice for each genotype. Mann–Whitney test, ***P ≤ 0.0001 and *P = 0.0038 compared with *Igsf21* ^+/+^mice. n.s., not significant. *Scale bars* represent 500 nm **a** and 200 nm **c**. Data are presented as mean ± s.e.m

Using electron microscopy, we further investigated the number of symmetric and asymmetric synapses in the stratum radiatum and the stratum pyramidale of the dorsal hippocampal CA1 region in *Igsf21* ^−/−^ mice (Fig. 6a, b). Generally, symmetric and asymmetric synapses correspond to inhibitory and excitatory synapses, respectively^38^. There was no difference in the number of symmetric or asymmetric synapses in the stratum pyramidale or stratum radiatum (Fig. 6a, b). This suggests that IgSF21 is dispensable for the formation of both inhibitory and excitatory synapses. To investigate whether IgSF21 plays a role in the organization of inhibitory presynaptic terminals, we next counted the number of total and docked SVs per bouton in symmetric synapses on dendrites in the stratum radiatum (Fig. 6c, d). Notably, the presynaptic boutons of symmetric synapses on dendrites in *Igsf21* ^−/−^ mice possessed far fewer total and docked SVs than those in *Igsf21* ^+/+^ littermates (*P* < 0.0001 for total SVs and *P* = 0.0001 for docked SVs) (Fig. 6c, d). In contrast, there was no difference in the number of total or docked SVs at presynaptic boutons of asymmetric synapses in the stratum radiatum of *Igsf21* ^−/−^ and *Igsf21* ^+/+^ littermates (Fig. 6c, d). In the presynaptic boutons of symmetric synapses on perisomatic regions in the stratum pyramidale, there was no difference in the number of total SVs (*P* = 0.24) but a slight decrease in the number of docked SVs (*P* = 0.0038) (Fig. 6c, d). Together, these results indicate that deleting IgSF21 impairs presynaptic organization of inhibitory, but not excitatory, synapses and preferentially affects those on dendrites in the stratum radiatum.

### Impaired inhibitory synaptic transmission in *Igsf21*^−/−^ mice

**Fig. 7:**
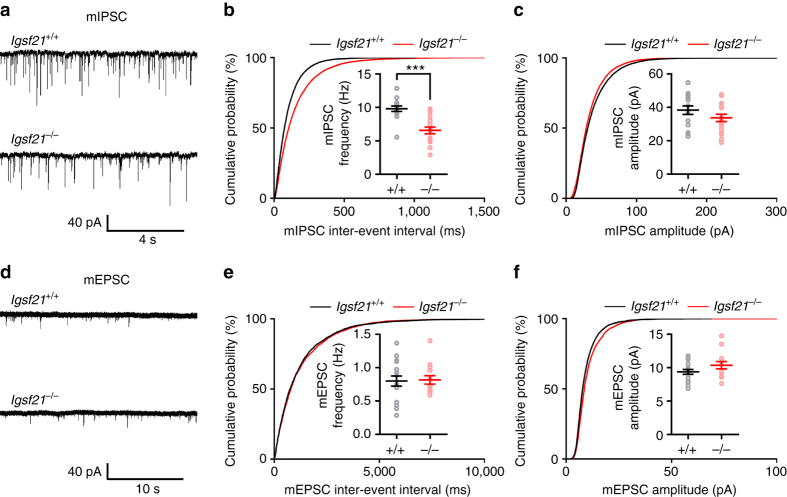
Inhibitory synaptic transmission is reduced in *Igsf21* ^−/−^ mice. **a**, **d** Representative recordings of mIPSCs **a** and mEPSCs **d** from hippocampal CA1 pyramidal neurons in acute slices from *Igsf21* ^+/+^ and *Igsf21* ^−/−^ mice. **b**, **c** Cumulative distributions of the mIPSC inter-event interval **b** and amplitude **c**. Insets in **b** and **c** display the mean frequency and amplitude (±s.e.m.), respectively. *n* = 15 cells from 7 *Igsf21* ^+/+^ mice and 17 cells from 7 *Igsf21* ^−/−^ mice. Unpaired *t*-test, ***P < 0.0001 for frequency and *P* = 0.1857 for amplitude. **e**, **f** Cumulative distributions of the mEPSC inter-event interval **e** and amplitude **f**. Insets in **e** and **f** display the mean frequency and amplitude (±s.e.m.), respectively. *n* = 16 cells from 7 *Igsf21* ^+/+^ mice and 13 cells from 9 *Igsf21* ^−/−^ mice. Unpaired *t*-test, *P* = 0.8658 for frequency and *P* = 0.1336 for amplitude. Data are presented as mean ± s.e.m

To investigate the effects of IgSF21 knockout on inhibitory synaptic function, we recorded miniature inhibitory and excitatory postsynaptic currents (mIPSCs and mEPSCs, respectively) from hippocampal CA1 pyramidal neurons in acute slices from *Igsf21* ^−/−^ and *Igsf21* ^+/+^ littermates (Fig. 7). mIPSC frequency was significantly decreased (corresponding to an increased inter-event interval) in *Igsf21* ^−/−^ mice relative to *Igsf21* ^+/+^ littermates (Fig. 7a, b). However, there was no difference in mIPSC amplitude in *Igsf21* ^−/−^ and *Igsf21* ^+/+^mice (Fig. 7a, c), as well as no difference in mEPSC frequency or amplitude (Fig. 7d–f). In principle, a decrease in mIPSC frequency could arise from a decrease in presynaptic GABA release probability, a decrease in the number of inhibitory synapses and/or, indirectly, a decrease in mIPSC amplitudes; however, these latter two possibilities can be eliminated as there was no change in inhibitory synapse number (Fig. 6a, b) or mIPSC amplitude (Fig. 7a, c). Thus, the reduced mIPSC frequency in *Igsf21* ^−/−^ mice suggests a decreased probability of presynaptic GABA release. Together with our observation of SV reduction in inhibitory presynaptic terminals in *Igsf21* ^−/−^ mice (Fig. 6c, d), these data indicate that IgSF21 regulates inhibitory synaptic transmission by controlling presynaptic function at inhibitory synapses.

### Impaired sensorimotor gating in *Igsf21*^−/−^ mice

**Fig. 8:**
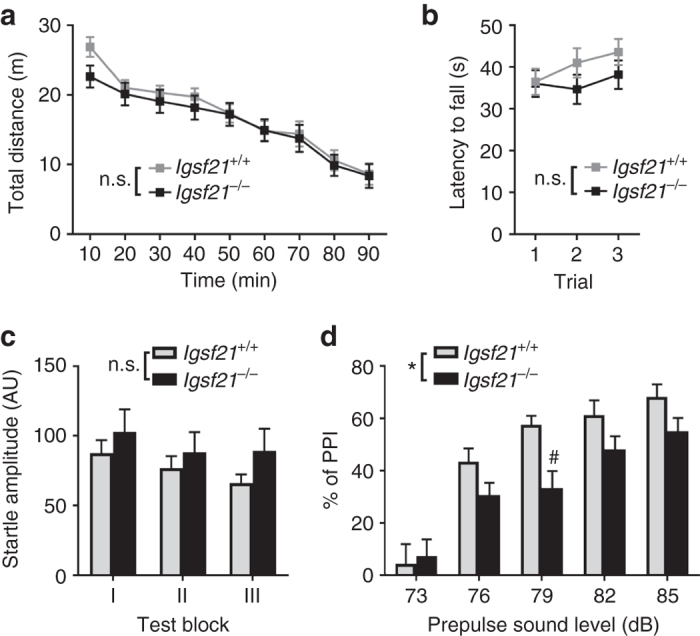
*Igsf21* ^−/−^ mice have a sensorimotor gating deficit. **a** There is no difference between *Igsf21* ^+/+^ and *Igsf21* ^−/−^ littermates in the total distance traveled during the open field test (total 90 min). *n* = 15 male mice from each genotype, two-way ANOVA, *F* (1, 252) = 2.199, *P* = 0.1393 between genotypes. n.s., not significant. **b** There is no difference between *Igsf21* ^+/+^ and *Igsf21* ^−/−^ littermates in latency to fall in the rotarod test. *n* = 15 male mice from each genotype, two-way ANOVA, *F* (1, 84) = 2.217, *P* = 0.1402 between genotypes. n.s., not significant. **c** There is no difference between *Igsf21* ^+/+^ and *Igsf21* ^−/−^ littermates in mean startle amplitudes in the pulse-alone trials for the three test blocks (Block I: 6 consecutive pulse-alone trials; Block II, 8 pulse-alone trials interspersed with prepulse+pulse trials; Block III: 6 consecutive pulse-alone trials). *n* = 15 male mice from each genotype, two-way ANOVA, *F* (1, 84) = 2.663, *P* = 0.1064 between genotypes. n.s., not significant. **d** *Igsf21* ^−/−^ mice display decreased pre-pulse inhibition (PPI). (Block II PPI, *n* = 15 male mice for each genotype, two-way ANOVA, *F* (1, 140) = 8.348, *P = 0.0045 between genotypes. ^#^ *P* = 0.0368 at 79 dB between the two genotypes by Bonferroni’s multiple comparisons test). Data are presented as mean ± s.e.m

To address whether IgSF21 is involved in brain functions, we performed behavioral assessment of sensorimotor processes in the *Igsf21* ^−/−^ mice by examining four general behaviors: open-field exploratory locomotion, rotarod performance, the acoustic startle reflex and prepulse inhibition (PPI) of the acoustic startle reflex, a measure of sensorimotor gating (the process of filtering out unimportant stimuli in the sensory environment)^39–41^. In the open field test, there was no difference in the total distance traveled by the *Igsf21* ^−/−^ and *Igsf21* ^+/+^ littermates (Fig. 8a). Also, *Igsf21* ^−/−^ and *Igsf21* ^+/+^ littermates had comparable latency to fall in the rotarod test (Fig. 8b). Thus, IgSF21 knockout had no effect on motor activity and coordination. In addition, *Igsf21* ^−/−^ and *Igsf21* ^+/+^ littermates displayed comparable amplitudes of the acoustic startle reflex (Fig. 8c). Interestingly, *Igsf21* ^−/−^ mice showed a significant reduction in PPI of the acoustic startle reflex (Fig. 8d). Thus, deleting IgSF21 impaired sensorimotor gating with no effect on motor activity and coordination.

## Discussion

In this study, we identified IgSF21 as a GPI-anchored membrane protein that selectively promotes inhibitory presynaptic differentiation. We further isolated NRX2α as an IgSF21-interacting presynaptic organizer through an unbiased proteomics screen. Our data showed that IgSF21 selectively interacts with NRX2α via its synaptogenic domain (Ig1) and recruits axonal NRX2α in a trans-interaction manner, providing molecular and cellular mechanisms by which IgSF21 induces inhibitory presynaptic differentiation through trans-synaptic adhesion. Our comprehensive analysis of *Igsf21* ^−/−^ mice revealed that in hippocampal CA1 pyramidal neurons, IgSF21 controls presynaptic organization of inhibitory synapses, especially those in the stratum radiatum, and that this control is crucial for normal GABA-mediated synaptic transmission. Accordingly, our behavioral experiments demonstrate that *Igsf21* ^−/−^ mice display impaired sensorimotor gating. Together, these data suggest that the IgSF21–NRX2α complex is essential for the differentiation of inhibitory presynaptic terminals on hippocampal CA1 pyramidal neurons and is indispensable for normal brain function.

Although numerous previous efforts have identified many synapse organizing complexes^19–31^, few of these regulate inhibitory synapse organization, either selectively or preferentially^29, 30, 32–34^. Here we show that IgSF21 promotes inhibitory, but not excitatory, presynaptic differentiation in cultured neurons, as Slitrk3 does^29^. However, in the hippocampal CA1 region, Slitrk3 knockout impairs inhibitory synapse organization in both the stratum radiatum and the stratum pyramidale^29^, while IgSF21 knockout impairs inhibitory synapse organization mainly in the stratum radiatum and very subtly in the stratum pyramidale. Knockout of NLG2, another inhibitory synapse organizer, impairs inhibitory synaptic organization in the hippocampal CA1 stratum pyramidale but not in the stratum radiatum^32^. Thus the most significant finding of this study is the identification of IgSF21 as a novel inhibitory synapse-specific organizer that works differently from previously identified inhibitory synapse organizers. The hippocampus^8^ and the cerebral cortex^11^ contain many different types of GABAergic interneurons whose axons tend to innervate specific subcellular compartments of target pyramidal neurons (the dendrite, the soma or the axon initial segment). Given that the stratum radiatum and the stratum pyramidale mainly contain dendritic and perisomatic inhibitory synapses on CA1 pyramidal neurons, respectively, IgSF21 may regulate presynaptic differentiation of inhibitory synapses innervated by specific subsets of GABAergic interneurons: those that mainly mediate dendritic inhibition. Examples of such interneurons include bistratified cells and Schaffer collateral-associated cells that preferentially innervate dendrites of CA1 pyramidal neurons in the stratum radiatum^8, 42^. Interneuron-subtype specificity for IgSF21 synaptogenic activity remains an interesting question, and future studies using methods such as artificial synapse formation assays in neuron cultures from conditional mutant mice with interneuron type-specific deletion of NRX2α would help clarify this important aspect of synapse organization.

The molecular basis for the selectivity of IgSF21 for inhibitory presynaptic differentiation may lie in its partner binding specificity. NLG2 and calsyntenin-3 have been characterized as NRX-binding synapse organizers that regulate inhibitory synapse development in the hippocampal CA1 region although calsyntenin-3 also regulates excitatory synapse development^35^. NLG2 binds both the α-isoform and β-isoform of NRX1/2/3^19^, whereas calsyntenin-3 binds only the α-isoform of NRX1/2/3^35^. In contrast, IgSF21 binds only NRX2α and no other isoforms. In coculture assays, NLG2 and calsyntenin-3 induce both inhibitory and excitatory presynaptic differentiation^27, 29, 35^ whereas IgSF21 induces inhibitory presynaptic differentiation exclusively. Similarly, while calsyntenin-3 knockout affects both excitatory and inhibitory synapse organization^35^, IgSF21 knockout selectively affects inhibitory synapse organization. These findings suggest that IgSF21 acts solely at inhibitory synapses due to its specific binding to NRX2α. Several NRX2α-based mechanisms that could underlie this specificity can already be eliminated. Restricted expression of NRX2α to GABAergic interneurons is an unlikely mechanism because NRX2α mRNA is expressed in all types of hippocampal neurons including pyramidal neurons^37^. Many molecular interactions with NRX depend on NRX-splicing^19, 22^; however, IgSF21 binds to NRX2α LNS1 regardless of NRX2α splice variation. Other less obvious mechanisms must be at play. For example, glutamatergic axons could express IgSF21 synaptogenic suppressors that bind to NRX2α. Or, GABAergic, but not glutamatergic, axons could express NRX2α-interacting coactivators of IgSF21 activity. Proteomics-based global identification of NRX2α-interacting proteins would be helpful to test these possibilities.

Our domain analyses revealed that IgSF21 Ig1 is indispensable for both IgSF21–NRX2α interaction and IgSF21 synaptogenicactivity. In contrast, IgSF21 Ig2 is dispensable for IgSF21–NRX2α interaction but is partly responsible for IgSF21 synaptogenic activity. Further, the IgSF21 splicing isoform with a shorter linker between Ig1 and Ig2 displays not quite half the synaptogenic activity of the IgSF21 isoform with a longer linker although both isoforms possess the exact same Ig1 and Ig2 domains. These findings suggest that synaptogenic activity of IgSF21 requires not only NRX2α binding (Ig1-dependent mechanism) but also other mechanisms that depend on Ig2 and the spliced linker. Synaptogenic activity of NLG1 requires NLG1 dimerization as well as NLG1–NRX interaction^43, 44^. In addition, synaptogenic activity of Slitrk1 requires not only Slitrk1–PTPδ interaction but also the lateral assembly of Slitrk1–PTPδ complexes^45^. Thus, the synaptogenic activity of IgSF21 may require IgSF21 dimerization or the lateral assembly of IgSF21–NRX2α complexes through its Ig2 domain and/or its linker. A future study of the crystal structure of the IgSF21–NRX2α complex will be needed to understand the structural basis for its synaptogenic activity.

A previous time-lapse imaging study has shown that inhibitory synapses exhibit coordinated organization of their presynaptic and postsynaptic sites^46^. However, our biochemical data show that hippocampal synaptosomes in *Igsf21* ^−/−^ mice contain decreased levels of VGAT but exhibit no significant reduction of the GABA_A_ receptor γ2 subunit or of gephyrin, a postsynaptic scaffold for GABA_A_ receptors^47^. Further, the hippocampal CA1 pyramidal neurons in *Igsf21* ^−/−^ mice have reduced mIPSC frequency but normal mIPSC amplitudes, indicating a reduction of quantal GABA release as a presynaptic effect of IgSF21. Our electron microscopy data also show that there is no reduction in the number of perisomatic and dendritic inhibitory synapses in *Igsf21* ^−/−^CA1 neurons but that these inhibitory synapses contain fewer total and/or docked SVs. These findings collectively suggest that inhibitory synapses *of Igsf21* ^−/−^ CA1 neurons have defective presynaptic release of GABA but normal functional GABA receptor density at their postsynaptic sites. The uncoordinated presynaptic and postsynaptic organization induced by IgSF21 knockout could be due to an imbalance in bidirectional trans-synaptic signaling via NRX2α: NRX2α acts as a receptor that mediates presynaptic organization signals induced by postsynaptic organizers such as NLGs^19, 22^, calsyntenin-3^35^ and IgSF21 but also acts as a ligand that induces postsynaptic organization, the signals of which are mediated by postsynaptic organizers such as NLGs^22, 48^. Further, IgSF21 binds to NRX2α at its LNS1 domain whereas NLGs binds to its LNS6 domain^19, 22^, suggesting that IgSF21 and NLGs regulate NRX2α functions through interacting with distinct domains of NRX2α. Thus, IgSF21 may play a role in balancing the bidirectional trans-synaptic roles of NRX2α for coordinated presynaptic and postsynaptic organization of inhibitory synapses by cooperating with other NRX2α-interacting organizers.

*Igsf21* is a vertebrate-specific gene with no paralogs and our data show its significant expression in the brain. Further *Igsf21* ^−/−^ mice exhibit sensorimotor gating deficits, which has also been demonstrated in several different mutant mouse lines with GABAergic signaling deficits^49–51^ and detected in patients with neuropsychiatric disorders, particularly schizophrenia^39–41^. These data suggest that IgSF21 may be involved in brain functions through the regulation of inhibitory presynaptic organization. Given that the ventral hippocampus is one of the brain regions involved in the regulation of sensorimotor gating^52^, impaired organization of GABAergic synapses in the ventral hippocampus may underlie the sensorimotor gating deficits of *Igsf21* ^−/−^ mice. Interestingly, deletion or alteration of the IgSF21-binding partner NRX2α in both mice and humans results in behavioral changes: NRX2α knockout mice exhibit autism-like and anxiety-like behaviors^53, 54^ and the human *NRXN2* gene encoding NRX2α is associated with ASD and schizophrenia^55^. Therefore, it would be important for future studies to more comprehensively characterize behavioral phenotypes of *Igsf21* ^−/−^ mice and/or region-specific *Igsf21* knockout mice and also to sequence the *IGSF21* gene locus in human patients with schizophrenia, ASDs and anxiety disorders.

In conclusion, we identified IgSF21-NRX2α as a novel synapse organizing complex specific to inhibitory synapses. In the hippocampal CA1 region, IgSF21 controls the organization of inhibitory presynaptic terminals mainly in the stratum radiatum. Our study thus identifies and characterizes a novel and unique molecular mechanism underlying inhibitory presynaptic organization, providing molecular insights into how complex GABA-mediated synaptic inhibition is established and regulated.

## Methods

### Plasmids

Rat IgSF21 (amino acid (aa) 1–468, NM_001271454.1) was isolated from a rat brain full-length cDNA library^23^ by performing a fibroblast-neuron coculture screen (Supplementary Fig. 1a, b). Mouse IgSF21 (aa 1–468, NM_198610.2) and IgSF21-S (aa 1–271, XM_006538799.3) were cloned by RT-PCR from mouse brain RNA extracts and subcloned into pBluescript II SK. For IgSF21-HA, an HA epitope coding sequence was inserted in frame 4 aa residues upstream of the putative C-terminal GPI anchor signal sequence. For mammalian expression controlled by the pCAG promoter, the coding region of the tagged constructs was subcloned into pCAG-EGFP (Addgene) between *Eco*RI and *Not*I, replacing the EGFP coding region. IgSF21 deletion constructs were generated by inverse PCR using mouse IgSF21-HA as the template to delete the regions encoding aa 31–141 (IgSF21ΔIg1) or aa 348–431 (IgSF21ΔIg2). A series of extracellularly HA-tagged neurexin (HA-NRX) constructs, HA-CD4 and HA-NLG2 were previously described^29, 56^. For the pDisplay-NRX2α constructs, the following coding regions of NRX2αS1–S4(+) were subcloned into pDisplay (Invitrogen): aa 29-199 (LNS1), aa 29-309 (LNS1-EGF1), aa 29-499 (LNS1-LNS2) and aa 29-680 (LNS1-LNS3). For IgSF21-Fc and NRX2α-Fc, the ectodomain regions of rat IgSF21 (aa 1–448) and mouse NRX2αS1–S4(+) (aa 1–1436) were subcloned into pc4-sp-Fc^27^ between the *Hin*dIII and *Eco*RI sites. All constructs were verified by DNA sequencing.

### Cell cultures, transfection and immunocytochemistry

Cultures of rat hippocampal and cortical neurons, COS-7 cells (ATCC) and HEK293 cells (ATCC), artificial synapse formation assay by coculture of COS-7 or HEK293 cells with neurons and immunocytochemistry were performed essentially as described^27, 29^. These cell lines were tested for mycoplasma contamination and experiments were performed under mycoplasma-free conditions.Transfections into COS-7 cells and HEK293 cells were performed using TransIT-LT1 (Mirus Bio. LLC). For transfections into hippocampal neurons, the ProFection Mammalian Transfection System (Promega) was used. Cultures were fixed with parafix solution (4% paraformaldehyde and 4% sucrose in phosphate-buffered saline (PBS) (pH 7.4)) for 12 min followed by permeabilization with PBST (PBS + 0.2% Triton X-100). The following primary antibodies were used for immunocytochemistry: anti-synapsin I (1:1000; rabbit; AB1543P; Millipore), anti-VGAT (1:1000; rabbit; 131 003; Synaptic Systems), anti-VGLUT1 (1:1000; guinea pig; AB5905; Millipore for coculture assays, or rabbit; 135 302, Synaptic Systems for IgSF21-HA localization assays, or mouse IgG1; 317G6, Synaptic Systems for bead assays), anti-gephyrin (1:1000; mouse IgG1;3B11, Synaptic Systems for coculture assays), anti-PSD-95 family (1:500; mouse IgG2a; 6G6-1C9, Thermo Scientific; recognizes PSD-95, PSD-93, SAP102 and SAP97), anti-Tau-1 (1:2000; mouse IgG2a; PC1C6, Millipore; recognizes dephosphorylated tau) and anti-HA (1:1000; mouse IgG2b; 12CA5, Roche for artificial synapse formation assays or 1:5000; rat IgG1; 3F10, Roche for colocalization analysis). In general, we used highly cross-adsorbed, Alexa-dye conjugated secondary antibodies generated in goat towards the appropriate species and monoclonal isotype (1:500; Alexa-488, Alexa-568 and Alexa-647; Invitrogen) for detection. But, for colocalization analysis using the rat anti-HA antibody, we used highly cross-adsorbed, Alexa-dye-conjugated secondary antibodies generated in donkey towards the appropriate species (1:500; Jackson ImmunoResearch). For labeling dendrites, we used anti-MAP2 (1:6000; chicken polyclonal IgY; ab5392; Abcam) revealed with AMCA-conjugated anti-chicken IgY (1:200; donkey IgG; 703–155–155; Jackson ImmunoResearch). To selectively label surface HA, cultures were fixed in parafix solution, blocked without permeabilization and then incubated with anti-HA antibody (1:1000) at 4 °C overnight. For the antibody uptake assay to measure recycling of SVs, live neurons were incubated with an antibody recognizing the luminal domain of synaptotagmin (1:500; mouse IgG1; clone 604.2; Synaptic Systems) for 30 min in culture medium at 37 °C in a 5% CO_2_ incubator. Images were acquired on a Leica DM6000 fluorescent microscope with a 63 × 1.4 numerical aperture (NA) oil objective and a Hamamatsu cooled CCD camera using Volocity software (Perkin Elmer). Images were acquired as 12-bit grayscale and prepared for presentation using Adobe Photoshop CS5. For quantification, sets of cells were stained simultaneously and imaged with identical settings. For IgSF21-HA colocalization analysis, images were acquired using a Leica FM6 B microscope with a 100 × 1.4 NA oil objective and a Hamamatsu C11440 ORCA-Flash 4.0 camera. Images were acquired as 16-bit grayscale and co-localization of IgSF21-HA puncta with VGLUT1 or VGAT puncta was analyzed by using Metamorph 7.8 software (Molecular Devices).

### PI-PLC digestion

PI-PLC digestions were performed essentially as described^57^. COS-7 cells were transfected with 2 μg of rat IgSF21-HA, washed with PBS 36 h post-transfection, and incubated in PBS or PBS containing PI-PLC (1 U ml^−1^; P-6466; Life technologies) for 1 h at 37 °C. After the incubation, the cells and the supernatants were collected. For immunoprecipitation of the IgSF21-HA from supernatants of cultured cells, the supernatant was incubated with anti-HA (1:300; mouse; clone HA-7; H3663; Sigma) for 2 h at 4 °C and then with Protein G sepharose (GE Healthcare) for 1 h at 4 °C. The proteins were separated by sodium dodecyl sulfate polyacrylamide gel electrophoresis in 12% gels and immunodetected using anti-HA antibody (1:2000; rabbit; ab9110; Abcam).

### Production of soluble Fc-fusion proteins

Based on previously described methods^27, 29^, soluble rat IgSF21 fused to Fc (IgSF21-Fc) and Fc for use as a negative control were generated using stable HEK293 cell lines transfected with the corresponding expression vectors with Zeocin-resistant cassettes and purified from culture media using Protein G sepharose beads. Soluble neurexin2α ectodomain fused to Fc (NRX2α-Fc) was generated by transient transfection into HEK293 cells.

### Proteomics screen for IgSF21-interacting proteins

Primary hippocampal neuron cultures were placed on cell culture dishes coated with poly-l-lysine at a density of 1.5 × 10^6^ cells per 60-mm dish. We coated 45 μl of Protein A magnetic beads (Dynabeads, Life Technology) with IgSF21-Fc or Fc protein. Coated beads were added to the cultured neurons in two 60 mm dishes at 14 days *in vitro*. After 24 h, cultures were washed once with D-PBS (including 0.9 mM CaCl_2_ and 0.49 mM MgCl_2_) and crosslinked with 1 mM 3,3ʹ-dithiobis (sulfosuccinimidyl propionate) (DTSSP; Pierce) in D-PBS. The crosslinked neurons were washed with D-PBS and incubated with lysis buffer containing 10 mM Tris-HCl, pH 8.0, 10 mM KCl, 5 mM EDTA, 5 mM EGTA, 0.6% Nonidet P-40 and protease inhibitors (complete EDTA-free; Roche). Bound proteins were purified by magnetic separation (DynaMag-2 Magnet; Life Technologies) and washed three times with lysis buffer followed by sonication. Bound proteins were further washed five times with wash buffer containing 50 mM Tris-HCl, pH 7.5, and 2% SDS followed by boiling at 98 °C for 5 min. Bound proteins were eluted by boiling in wash buffer containing 5% 2-mercapto-ethanol, separated by SDS-PAGE and stained with silver staining for in-gel digestion. The gel lanes were excised into 16 individual fractions. Proteins in each fraction were reduced, alkylated and digested with trypsin as described^58^. The resulting peptides were analyzed by liquid chromatography/tandem mass spectrometry using an LTQ OrbitrapVelos instrument (ThermoFisher Scientific, Bremen, Germany) equipped with a Proxeonnanoelectrospray ion source. For protein identification, Mascot 2.5 software (Matrix Science) was used to search the rat Uniprot database. The mass spectrometry proteomics data have been deposited to the ProteomeXchange Consortium via the PRIDE^59^ partner repository with the dataset identifier PXD006622 and 10.6019/PXD006622.

### Cell surface binding assay

Cell surface binding assays were performed essentially as described^27, 29^. COS-7 cells were transfected with the expression vectors for candidate binding proteins and maintained for 24 h. The transfected COS-7 cells were washed with extracellular solution (ECS, containing 168 mM NaCl, 2.4 mM KCl, 20 mM HEPES (pH 7.4), 10 mM d-glucose, 2 mM CaCl_2_ and 1.3 mM MgCl_2_) that contained 100 μg ml^–1^ bovine serum albumin (BSA) and then incubated with ECS and BSA containing 100 nM purified Fc-fusion protein for 1 h at room temperature. These cells were washed in ECS, fixed with parafix solution, incubated with blocking solution followed by rabbit anti-HA without permeabilization and then immunostained with Alexa488-conjugated goat anti-rabbit IgG (H+L) (1:500; Invitrogen) and Alexa594-conjugated donkey anti-human IgG (H+L) (1:500; Jackson ImmunoResearch) to label surface HA and bound Fc proteins, respectively. Images were acquired on a Leica DM6000 fluorescent microscope with a 40 × 0.75 NA dry objective and a Hamamatsu cooled CCD camera using Volocity software (Perkin Elmer).

### Generation of *Igsf21*^−/−^ mice

To generate *Igsf*21 mutant mice, ES cell clones (Igsf21^tm2a(EUCOMM)Hmgu^) were obtained from the European Conditional Mouse Mutagenesis Consortium (Eucomm). In this cell line, the targeting trapping cassette containing the Engrailed-2 splice acceptor upstream of β-geo reporter is inserted before a floxed *Igsf*21 exon 4. The reporter creates an N-terminal IgSF21 truncation that does not have any immunoglobulin-like domains. Homologous recombination in the obtained ES cells was verified by Southern blotting. Clone F07 was injected into C57BL/6J blastocysts to generate chimeras in the same B6 genetic background, and underwent germline transmission by crossing with C57BL/6J mice to produce the *Igsf21* ^−/−^ line. *Igsf21* ^−/−^ mice were bred into the C57BL/6 background. Genotyping of *Igsf21* ^−/−^mice was performed using the following two primers: P1 (5′-TCA GAC AAT GGT CCC TAT GAG-3′) and P2 (5′-CTC ATG GCA CTT GCT CCT TG-3′). The expected size of the PCR products for wild type and mutant genes is 632 and 697 bp, respectively. Littermates derived from heterozygous parents were used in this study. All animal experiments were carried out in accordance with the Canadian Council on Animal Care guidelines and approved by the IRCM Animal Care Committee. Mice were group-housed (two to five per cage) and maintained on a 12-h light/dark cycle (lights on 6:00–18:00).

### Synaptosome and PSD preparations and immunoblotting

Purification of synaptic fractions consisting of the crude synaptosome, synaptosome and PSD fractions from 3- to 4-week-old *Igsf21* ^+/+^ and *Igsf21* ^−/−^ mice of mixed sex was performed as described previously^23^. For all samples, protein concentrations were measured in DC protein assays (Biorad). After normalizing protein concentration, samples were run on 10 or 12% polyacrylamide gels. Gels were transferred onto Immobilon P membranes (Millipore). Membranes were blocked in 5% skim milk and 0.1% Triton X-100 in PBS and incubated with one of the following primary antibodies: anti-IgSF21 (1:1000; rabbit; 21465-1-AP; Proteintech), anti-VGAT antibody (1:10,000; rabbit; 131 003; Synaptic Systems), anti-VGLUT1 (1:5000; rabbit; 135 302; Synaptic Systems), anti-gephyrin (1:10,000; mouse IgG1; clone 3B11; 147 111; Synaptic Systems), anti-GABA_A_γ2 (1:3000; rabbit; 224 003; Synaptic Systems), anti-GABA_A_α1 (1:100; mouse IgG2a; clone N95/35 supernatant; 73-136; NeuroMab), anti-PSD-95 family (1:2000; mouse IgG2a; clone 6G6-1C9; MA1-045; Thermo Scientific), anti-SynGAP (1:2000; rabbit; 06-900; Millipore (Upstate)), anti-GluA1 (1:500; mouse IgG1; clone N355/11; 75-327; NeuroMab), anti-GluN2A (1:1000; rabbit; 07-632; Millipore), anti-GluN2B (1:500; mouse IgG2b; clone N59/36; 75-101; NeuroMab), anti-neuroligin1 (1:300; mouse IgG1; clone N97A/31; 75-160; NeuroMab), anti-neuroligin2 (1:3000; mouse IgG1; clone 5E6; 129 511; Synaptic Systems), anti-neuroligin3 (1:300; mouse IgG1; clone N110/29; 75-158; NeuroMab), anti-synaptophysin (1:10,000; mouse IgG1; clone SY38; MAB5258; Millipore), anti-synapsin I (1:2000; rabbit; AB1543P; Millipore), anti-neuronal class III β-Tubulin (1:2000; mouse IgG2a; clone Tuj1;801201; Biolegend), anti-GFAP (1:2000; Rabbit; Z0334; Dako) and anti-β-actin (1:2000; rabbit; ab8227; Abcam). Membranes were washed with PBS containing 0.1% Triton X-100 and incubated with one of the following secondary antibodies: HRP-conjugated anti-mouse IgG (H+L) (1:10,000; 115-035-146; Jackson ImmunoResearch) or HRP-conjugated anti-rabbit IgG (H+L) (1:10,000; 111-035-144; Jackson ImmunoResearch). Signals were developed using Immobilon Western Chemiluminescent HRP Substrate (Millipore) and captured by ImageQuant LAS 4000 (GE Healthcare). Band signal intensity was measured using Metamorph 7.8 software (Molecular Devices) and normalized to β-actin signal intensity for quantification.

### Immunohistochemistry

Immunofluorescence of brain sections was performed on methanol-fixed fresh frozen brain sections. We anesthetized 4-week-old *Igsf21* ^+/+^ and *Igsf21* ^−/−^ mouse littermates of mixed sex with ketamine/xylazine and killed them by transcardial perfusion with PBS (pH 7.4) for blood removal. The brain was removed from the skull, frozen in O.C.T. compound (Tissue-Tek) on dry ice and then stored at −80 °C until sectioning. Coronal brain sections (10 µm thick) were cut using a cryostat (Thermo Scientific, CryoStar NX70). The sections were mounted on Superfrost Plus slides and stored at −80 °C. Slides were submerged in a −20 °C methanol bath for 15 min, then washed three times for 5 min each with PBS, blocked for 1 h at room temperature with blocking solution (PBS containing 5% BSA (wt/vol), 2.5% normal goat serum (vol/vol), 2.5% normal donkey serum (vol/vol)) and 0.25% Triton X-100 (vol/vol)). The sections were subsequently incubated overnight at 4 °C with combinations of the following primary antibodies: anti-VGAT antibody (1:500; rabbit; 131 003; Synaptic Systems), anti-VGLUT1 (1:2000; guinea pig; AB5905; Millipore), anti-gephyrin (1:1000; mouse; 141 111; Synaptic Systems) and anti-NeuN (1:500; mouse; clone A60; Millipore). After three washes with PBS, the sections were incubated for 1 h at room temperature with combinations of the following highly cross-absorbed secondary antibodies: Alexa488-conjugated donkey anti-rabbit IgG (1:500; 711-545-152; Jackson ImmunoResearch), Alexa594-conjugated donkey anti-guinea pig IgG (1:500; 706-585-148; Jackson ImmunoResearch), Alexa594-conjugated donkey anti-mouse IgG (1:500; 715-585-150; Jackson ImmunoResearch) and Alexa647 goat anti-mouse IgG (1:500; A21236; Life technologies). After washing with PBS, the sections were sealed with a mounting reagent (5% DABCO (wt/vol) and 90% glycerol in 0.02 M Na_2_HPO_4_). Confocal images were taken sequentially using a Leica SP8 microscope with 488, 552 and 638 nm lasers and custom filter sets with a 5 × 0.15 NA dry objective for low-magnification images and a 40 × 1.30 NA oil-immersion objective for high-magnification images. 12-bit grayscale images were acquired using Leica Application Suite (LAS) X software at a scan speed of 400 Hz, and the image dimensions were 2048 × 2048 pixels with a pixel size of 1.138 μm (5×) or 0.142 μm (40×) and an optical thickness of 51.43 μm (5×) or 1.04 μm (40×).

### X-gal staining

Four-week-old *Igsf21* ^*+/*−^and *Igsf21* ^+/+^ littermates were anesthetized and killed by transcardial perfusion with PBS (pH 7.4) and 4% paraformaldehyde in PBS. After perfusion, the brain was removed, post-fixed for 3 h in 4% paraformaldehyde in PBS at 4 °C and cryoprotected in 30% (w/v) sucrose in PBS for 24 h at 4 °C. The brains were frozen in O.C.T. compound (Tissue-Tek) on dry ice. Sagittal brain sections (50 μm thick) were cut using a cryostat (Thermo Scientific, CryoStar NX70). Free-floating sections were fixed with 0.2% glutaraldehyde/PBS for 5 min, washed three times with PBS and then incubated in 5-bromo-4-chloro-3-indolyl β-d-galactopyranoside (X-gal) working solution (1 mM MgCl_2_, 3 mM K_4_[Fe(CN)_6_]•3H_2_O, 3 mM K_3_[Fe(CN)_6_], 0.1% Triton X-100, 1 mM X-gal) at 37 °C for 24 h. Sections were washed with 1 mM EDTA/PBS solution to stop the reaction and sealed with the mounting reagent described above. Images were acquired using a Leica M165FC microscope with a ×0.63 PlanAPO objective and a Leica DFC450C digital camera using LASv4.3 software. For nuclear counterstaining, slices were stained with X-gal and then incubated with DAPI (100 ng ml^–1^) in PBS for 30 min at room temperature. Images co-stained with X-gal and DAPI were acquired using a Leica FM6 B microscope using a DFC480 camera for the color image and a Hamamatsu C11440 ORCA-Flach 4.0 camera for the fluorescent signal.

### Electron microscopy

Twelve littermates (6 *Igsf21* ^+/+^ and 6 *Igsf21* ^−/−^, 4 weeks of age, mixed sex) were anesthetized and perfused transcardially with 4% paraformaldehyde and 1% glutaraldehyde in 100 mM phosphate buffer (pH 7.4) for 15 min. Brains were removed and post-fixed in the same buffer overnight at 4 °C. The small pieces (1 × 1 × 1.5 mm) of hippocampus including the CA1 stratum pyramidale and stratum radiatum were dissected and fixed with 2.5% glutaraldehyde and 4% sucrose in 0.1 M sodium cacodylate buffer overnight at 4 °C and washed with 0.1 M cacodylate buffer for 1–2 h, followed by post-fixation with 1% aqueous OsO_4_ and 1.5% aqueous potassium ferrocyanide in the same buffer for 2 h at 4 °C. After dehydration with increasing concentrations of acetone, the samples were embedded in Epon812. To verify the location within the samples of the stratum pyramidale or the stratum radiatum, 500-nm-thick sections were prepared with an ultra-microtome and stained with toluidine blue. Ultrathin sections (90–100 nm thick) were cut using an ultra-microtome and stained with 4% uranyl acetate and Reynold’s lead. Images of the CA1 stratum pyramidale (> 30) and stratum radiatum (> 50) were obtained using an FEI Tecnai 12 BioTwin 120 kV transmission electron microscope equipped with an AMT XR80C CCD Camera System (Philips). The final magnification of the captured images was ×31,330. To quantify synapse number, we combined 10 images as one field for the stratum radiatum and 6 images as one field for the stratum pyramidale. Symmetric and asymmetric synapses and total and docked SVs per bouton were visually identified and counted using Metamorph 7.8. SVs observed to be in physical contact with the presynaptic plasma membrane were defined as docked vesicles. The SV number data were derived from 117 symmetric and 124 asymmetric synapses in dendritic regions in the *Igsf21* ^+/+^ stratum radiatum, 117 symmetric and 123 asymmetric synapses in dendritic regions in the *Igsf21* ^−/−^ stratum radiatum, and 111 and 116 symmetric synapses in perisomatic regions in the *Igsf21* ^+/+^ and *Igsf21* ^−/−^stratum pyramidale, respectively (17–25 synapses per mouse, 6 mice for each genotype). All images were acquired and analyzed while blind to genotype.

### Electrophysiology

We used 3- to 4-week-old littermate mice of mixed sex for acute hippocampal slice electrophysiology. Mice were anesthetized and decapitated, and their brains were rapidly removed and placed in ice-cold dissection solution saturated with 95% O_2_ and 5% CO_2_ and containing (in mM) 87 NaCl, 2.5 KCl, 1.25 NaH_2_PO_4_, 7.0 MgCl_2_, 0.5 CaCl_2_, 25 NaHCO_3_, 25 glucose, 1 ascorbic acid, 3 pyruvic acid and 75 sucrose (295 mOsmol). A block of tissue containing the hippocampus was prepared and transverse hippocampal slices (300 μm thick) were cut in ice-cold dissection solution using a vibratome (Leica VT1000S). Slices were transferred to a prechamber (Warner Instruments, BSC-PC) filled with oxygenated artificial cerebrospinal fluid (ACSF) containing (in mM) 126 NaCl, 2.5 KCl, 1.25 NaH_2_PO_4_, 2 MgCl_2_, 2 CaCl_2_, 26 NaHCO_3_ and 10 glucose (295–305 mOsmol), allowed to recover first for 30 min at 30 °C and then at room temperature for 1 h. For recording, slices were transferred to a submerged recording chamber continuously perfused (2 ml min^−1^) with oxygenated ACSF maintained at 28 ± 0.5 °C. For mEPSC recordings, CA1 pyramidal neurons were voltage clamped at −70 mV. Recording pipettes (4–6 MΩ) were filled with solution containing (in mM) 130 Cs-methanesulfonate, 5 CsCl, 2 MgCl_2_, 10 EGTA, 10 HEPES, 5 phosphocreatine 2 ATP(tris) and 0.4 GTP (tris), with the pH adjusted to 7.2–7.4 by CsOH(~290 mOsmol). During mEPSC recording, tetrodotoxin (TTX; 500 nM; Alomone) and gabazine (20 μM; Tocris) were added to the ACSF to block action potentials and GABA_A_ receptor-mediated inhibitory synaptic currents, respectively. For mIPSC recordings, CA1 pyramidal neurons were voltage clamped at −70 mV and the recording pipette was filled with solution containing (in mM) 125 CsCl, 0.1 CaCl_2_, 1 MgCl_2_, 10 HEPES, 10 EGTA, 4 ATP (Mg^2+^ salt), 0.3 GTP (Na^+^ salt), with the pH adjusted to 7.2–7.4 using CsOH and the osmolality adjusted to ~290 mOsmol. The recording solution used to isolate mIPSCs contained NBQX (10 µM; Tocris), DL-AP5 (50 µM; Tocris) and TTX (500 nM; Alomone) to block excitatory synaptic transmission and action potentials. Synaptic currents were recorded using an EPC-10 patch-clamp amplifier (HEKA Electronics), filtered at 2.9 kHz with a Bessel filter and digitized at 10 kHz. The frequency and amplitude of mEPSCs and mIPSCs were analyzed using MiniAnalysis software (Synaptosoft Inc.). For all experiments and analysis, the experimenter was blind to genotype.

### Behavioral experiments

All behavioral experiments were approved by both the McGill University Animal Care Committee and the IRCM Animal Care Committee and performed between 8:00 and 18:00 by experimenters blind to genotype. The same group of mice (15 *Igsf21* ^+/+^ and 15 *Igsf21* ^−/−^ male littermates at 6–11 postnatal weeks) was used in all behavioral experiments. Mice were maintained in a 12-h light/dark cycle (lights on 8:00–20:00 at the Douglas Mental Health University Institute) in a room at 19–22 °C. Data analysis for all tests was also performed while blind to genotype.

For open field test, locomotor activity boxes containing infrared sensors (AccuScan Instruments, Columbus, OH, USA) were adapted for use with mice by placing inserts that divide the space into four equal parts (20 × 20 × 29.5 cm). Mice were placed in the center of the chamber and allowed to explore freely for a total of 90 min. To assess locomotor activity, the total distance (cm) travelled in each 10-minute period was measured.

Rotarod testing was performed using a five-station rotarod treadmill (Model LE-8500, Harvard Apparatus Canada). Mice were placed on a rotating rod and were first trained on the apparatus for five 2-min trials at an interval of 10 min and a constant speed of 4 r.p.m. After the training, three test trials were performed. During the test trials, the speed of rotation was gradually increased at 0.3 r.p.m. per second from 4 to 40 r.p.m. Trials terminated when the animals fell from the rotating rod or after a maximum time of 5 min. The mean over the three test trials of the time spent on the rod was taken as the measure of performance in this test.

Testing of acoustic startle reflex and PPI was conducted in eight startle chambers (San Diego Instruments, San Diego, CA, USA) each consisting of a Plexiglas chamber (8 cm diameter, 16 cm long) mounted on a Plexiglas base within a lit, ventilated and sound-attenuating chamber. A speaker mounted on the ceiling of the chamber provided the background noise (70 dB) and the prepulse and pulse stimuli. A piezoelectric strain meter on the base transduced the startle response. Stabilimeter readings were rectified, digitized on a 4095 scale and recorded by a computer. An average of 100 1-ms readings beginning at stimulus onset was used to measure the startle amplitude. To assess the startle amplitude without a prepulse, a 120 dB, 30-ms stimulus was presented alone. To assess PPI, this startle stimulus was preceded by a 30-ms prepulse stimulus at one of 73, 76, 79, 82 or 85 dB. Each session consisted of a 5-min acclimation period followed by three blocks of trials. Blocks I and III each consisted of 6 startle trials (no prepulse was presented). Block II consisted of 38 trials: 8 startle trials, 5 PPI trials at each of the 5 prepulse intensities and 5 “null” trials in which no stimuli were presented. These trials were performed with a variable inter-trial interval (5–30 s, average of 15 s) in random order, with the restriction that no more than 2 trials of the same type occurred sequentially. For data analysis, we took the average of the 8 startle trials presented during Block II for each subject to measure the startle response. We also averaged the 5 PPI trials at each of the 5 different prepulse intensities, and then expressed these values as a percentage of the average response for the 8 startle trials using the following formula: [(startle-PPI)/startle] × 100. We also took the averages of the 6 startle trials in Block I and Block III to detect genotype-dependent differences in habituation.

### Quantitative fluorescence

All imaging and image analysis was done while blind to the experimental condition. All the data for imaging were collected in random order. Analysis was performed using Metamorph 7.8. For coculture assays, fields for imaging were selected based on the surface HA and phase contrast channels to detect HA-positive HEK293 cells in a neurite-rich region without considering the other fluorescence channels. The fluorescence channels corresponding to presynaptic markers were thresholded and the total intensity of puncta within all regions positive for both surface HA (labeling transfected HEK293 cells) and dephospho-tau (labeling axons) but negative for MAP2 (labeling dendrites) was measured. For IgSF21-HA localization analysis, images from the IgSF21-HA and presynaptic marker channels were thresholded separately to isolate appropriate puncta. The association between the presynaptic markers and IgSF21-HA puncta was defined as the pixel overlap of the thresholded IgSF21-HA image with the thresholded image of each presynaptic marker. For cell surface binding assays, after off-cell background was subtracted, the average intensity of bound Fc protein per COS-7 cell area was measured and normalized to the average intensity of surface HA signal. For quantitative analysis of brain slices, we determined regions of interest (ROIs) using NeuN images. Quantification of VGAT and VGLUT1 puncta intensity in the stratum radiatum was performed in the same ROI. VGAT and VGLUT1 images were thresholded by a constant grayscale value equal to the average of the automatically calculated threshold level of all analyzed images. We measured the total intensity of all VGAT or VGLUT1 puncta in each ROI (total intensity (AU) per μm^2^) and normalized the intensity values to the mean value of wild-type control.

### Statistical analysis

Statistical comparisons were made using GraphPad Prism 6. Data with normal distributions were analyzed by parametric tests, whereas data with non-normal distributions were analyzed by non-parametric tests. For parametric tests, statistical differences between two groups were calculated with an assumption of similar variance using two-tailed unpaired or paired *t*-tests, and between more than two groups using one-way ANOVA with *post hoc* Tukey’s multiple comparisons tests or two-way ANOVA with *post hoc* Bonferroni’s multiple comparisons tests, as indicated in the figure legends. For non-parametric tests, two-tailed Mann–Whitney tests and Kruskal–Wallis one-way ANOVA with *post hoc* Dunn’s multiple comparisons tests were used to compare two groups and more than two groups, respectively, as indicated in the figure legends. Our sample sizes were determined based on previous studies in the field. All data are reported as the mean ± standard error of the mean (s.e.m.) from three independent experiments and statistical significance was defined as *P* < 0.05.

### Data availability

The data that support the findings of this study are available within the article and its Supplementary Information file, or from the corresponding author upon request. All raw mass spectrometry proteomics data are available via ProteomeXchange (http://www.proteomexchange.org/) with identifier PXD006622 and 10.6019/PXD006622.

## Electronic Supplementary Material

Supplementary Information
