# Supplementary material for: IgSF21 promotes differentiation of inhibitory synapses via binding to neurexin2α
Source: Nat Commun. 2017 Sep 1;8:408. doi: 10.1038/s41467-017-00333-w (PMC5581337; doi:10.1038/s41467-017-00333-w)
Supplement: Supplementary file 1 — Supplementary Information [file 41467_2017_333_MOESM1_ESM.pdf]

File Name: Supplementary Information
Description: Supplementary Figures.

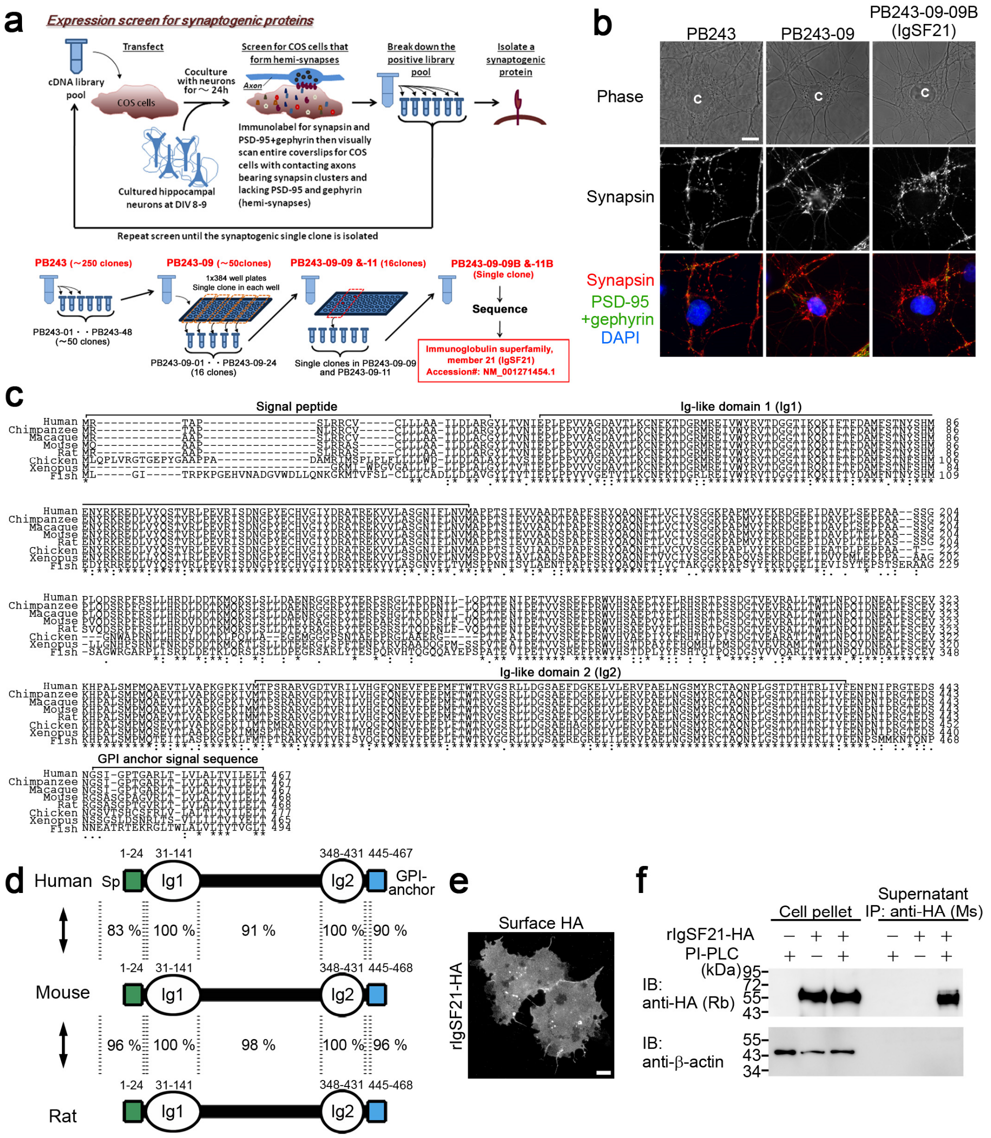

Supplementary Figure 1. Identification, protein alignment, and GPI-anchor modification of IgSF21

(**a**) Flow diagram illustrating the experimental protocol for an unbiased expression screen based on an artificial synapse formation assay to identify novel synaptogenic molecules. Using a rat brain full-length cDNA library in this screen isolated IgSF21.

(**b**) Example fluorescence images of the co-cultures from the screen in (**a**) showing results from positive pools. C, COS-7 cells transfected with indicated pools or clone. The scale bar represents 20 μm.

(**c**) Protein sequences of IgSF21: human (NP_116269.3), chimpanzee (XP_003307882.2), macaque (XP_011742106.1), mouse (NP_941012.1), rat (NP_001258383.1), chicken (XP_417520.3), xenopus (NP_001086244.2), and fish (XP_008407341.1). All of them possess an N-terminal signal peptide, two immunoglobulin-like domains (Ig1 and Ig2), and a putative C-terminal GPI-anchor signal sequence but no transmembrane domain.

(**d**) The primary structure and the percentage of identical amino acid (aa) residues of human, mouse, and rat IgSF21. Their Ig1 and Ig2 domains are 100% identical. Sp, signal peptide.

(**e**) Surface HA immunostaining of rat IgSF21 tagged with HA (rIgSF21-HA) in COS-7 cells. IgSF21 is expressed on the cell surface. The scale bar represents 20 μm.

(**f**) IgSF21 is a GPI-anchored protein. COS-7 cells expressing rIgSF21-HA were incubated with phosphatidylinositol-specific phospholipase C (PI-PLC). Processed rIgSF21-HA in the supernatant was collected by immunoprecipitation using mouse (Ms) anti-HA antibody and then detected by immunoblot using rabbit (Rb) anti-HA antibody. IP, immunoprecipitation; IB, immunoblot.

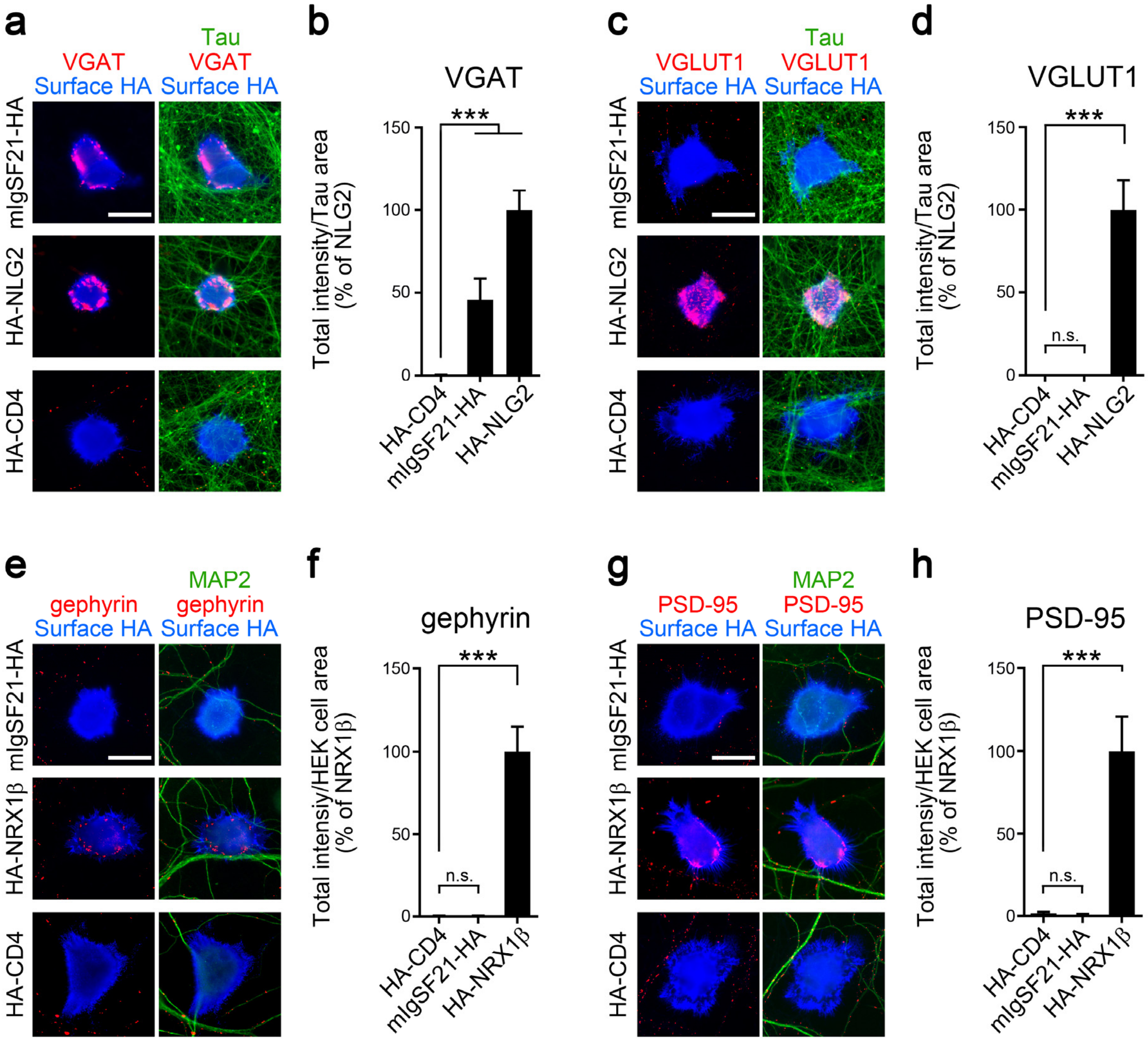

Supplementary Figure 2. Artificial synapse formation assays for IgSF21 using cortical neuron cultures, and testing for post-synaptic induction activity of IgSF21 using hippocampal neuron cultures

(**a**,**c**) HEK293 cells expressing mouse IgSF21 tagged with HA (mIgSF21-HA) induce VGAT (**a**) but not VGLUT1 (**c**) clustering along contacting axons in cortical neuron cultures at 10 days *in vitro* in the coculture assay. HEK293 cells expressing the HA-neuroligin2 (HA-NLG2) positive control, but not those expressing the HA-CD4 negative control, induce both VGAT and VGLUT1 clustering in the coculture assay.

(**b**,**d**) Quantification of the total integrated intensity of VGAT (**b**) and VGLUT1 (**d**) associated with HEK293 cells expressing the indicated HA-tagged proteins. n > 25 cells each from three independent experiments, Kruskal-Wallis one-way ANOVA, $P < 0.0001$ for VGAT and $P < 0.0001$ for VGLUT1. ***$P < 0.0001$ compared with HA-CD4 by Dunn's multiple comparisons test. n.s., not significant.

Scale bars represent 20 μm. Data are presented as mean ± s.e.m.

(**e**,**g**) HEK293 cells expressing mouse IgSF21 tagged with HA (mIgSF21-HA) do not induce gephyrin (**e**) or PSD-95 (**g**) clustering on dendrites (labeled with MAP2) in hippocampal neuron cultures at 15 days *in vitro*. As a positive control, HA-neurexin (NRX)1βS4(-) expressed in HEK293 cells induces gephyrin and PSD-95 clustering in the coculture assay.

(**f**,**h**) Quantification of the total intensity of gephyrin (**f**) and PSD-95 (**h**) associated with HEK293 cells expressing the indicated HA-tagged proteins. n > 30 cells each from three independent experiments, Kruskal-Wallis one-way ANOVA, $P < 0.0001$ for gephyrin and $P < 0.0001$ for PSD-95. ***$P < 0.0001$ compared with HA-CD4 by Dunn's multiple comparisons test. n.s., not significant.

Scale bars represent 20 μm. Data are presented as mean ± s.e.m.

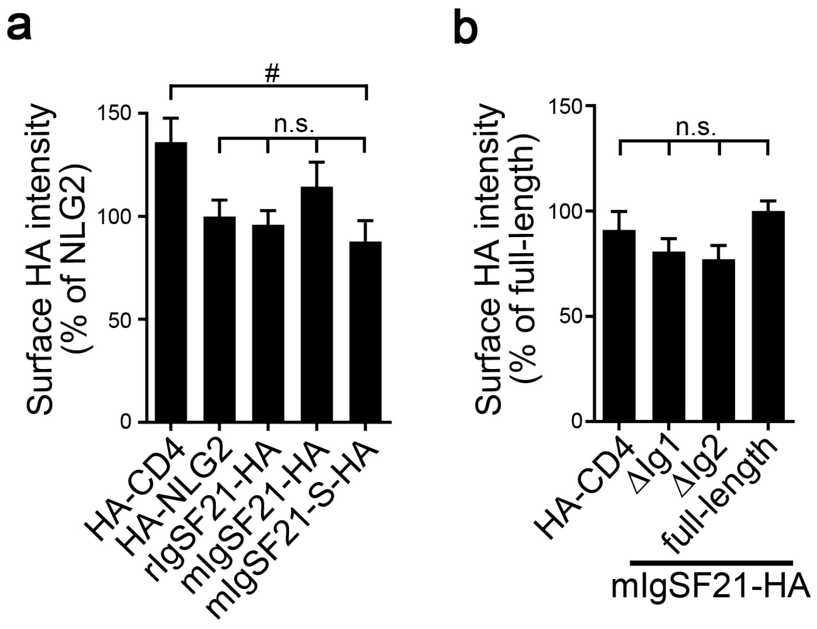

Supplementary Figure 3. No significant difference in surface expression level of the IgSF21-HA constructs used in artificial synapse formation assays

(**a**) There is no significant difference in surface HA intensity on HEK cells transfected with the IgSF21 constructs used for the quantification shown in Figure 1c. Kruskal-Wallis one-way ANOVA, P = 0.0227. $^{\#}P$ = 0.0174 between HA-CD4 and mIgSF21-S-HA by Dunn's multiple comparisons test. n.s., not significant.

(**b**) There is no significant difference in surface HA intensity on HEK cells transfected with the IgSF21 constructs used for the quantification shown in Figure 1i. Kruskal-Wallis one-way ANOVA, P = 0.1169 followed by Dunn's multiple comparisons test. n.s., not significant.

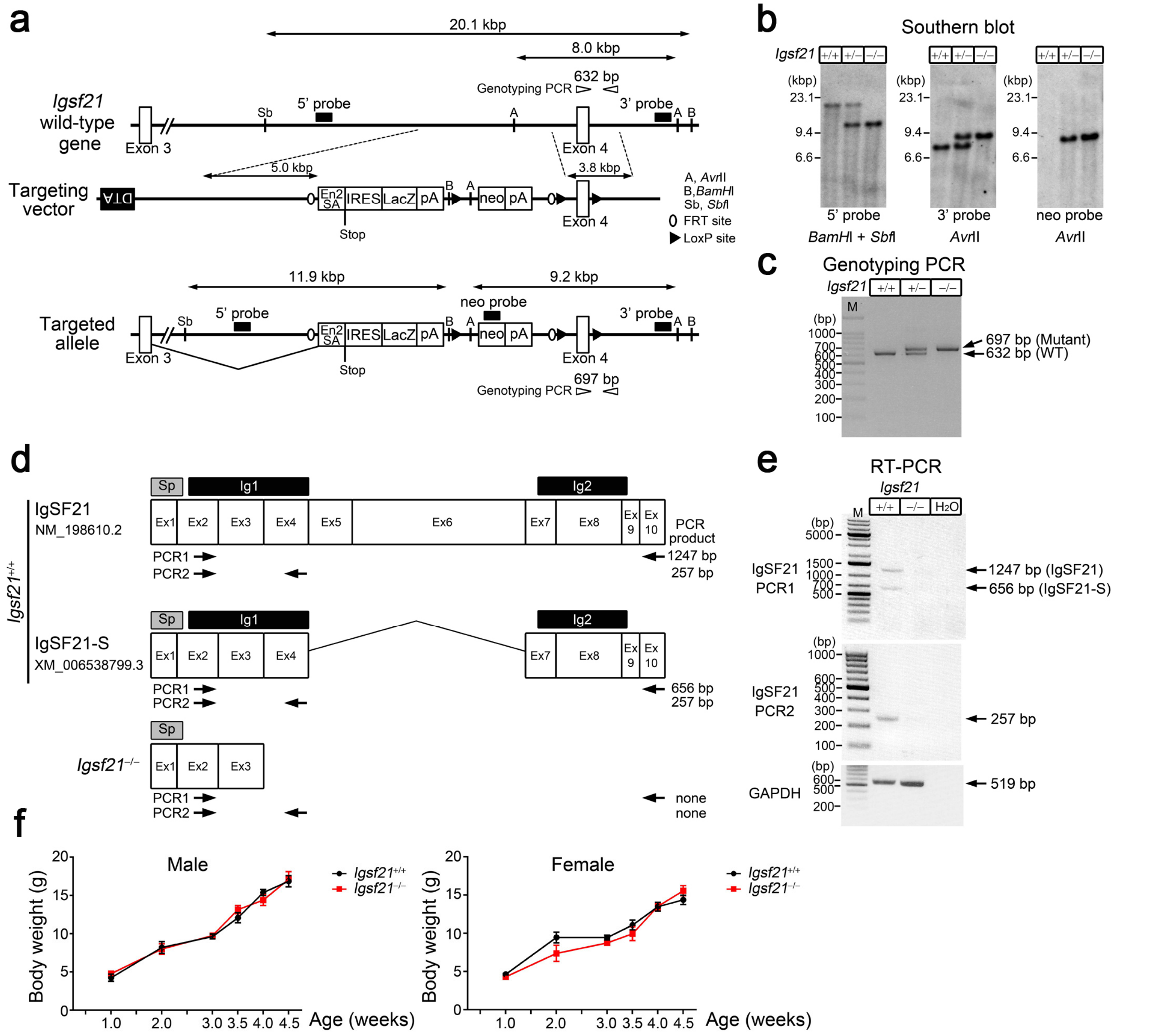

Supplementary Figure 4. Generation of *Igsf21* mutant mice

(**a**) Gene targeting to generate an *Igsf21* mutant mouse by a gene trapping strategy. The trapping cassette containing the engrailed-2 splice acceptor (En2SA) upstream of β-geo reporter (LacZ) is inserted before *Igsf21* exon 4 which is flanked by LoxP sites. neo, a neomycin-resistant gene cassette; DTA, a diphtheria toxin gene; IRES, an internal ribosome entry site; pA, SV40 polyadenylation site.

(**b**) Southern blot analysis using 5' and 3' outside probes and neo probes to verify the expected homologous recombination.

(**c**) PCR results of mouse genotyping. The expected sizes of the PCR products from the wild-type and targeted alleles are 632 and 697 bp, respectively. M, molecular weight marker.

(**d,e**) Exon structure of mRNAs encoding mouse IgSF21 (NM_198610.2), IgSF21-S (XM_006538799.3) and the targeted gene product. Arrows indicate primer positions for RT-PCR. RT-PCR results show disrupted transcription of IgSF21 and IgSF21-S in *Igsf21*$^{-/-}$ mice. Sp, signal peptide; Ig1 and Ig2, the first and second immunoglobulin-like domains. Ex, exon; M, molecular weight marker.

(**f**) Body weight analysis of *Igsf21*$^{+/+}$ and *Igsf21*$^{-/-}$ mice shows normal growth of *Igsf21*$^{-/-}$ mice. > 50 mice for each sex and genotype, two-way ANOVA, P = 0.7441 between genotypes for male and P = 0.1575 between genotypes for female. Data are presented as mean ± s.e.m.

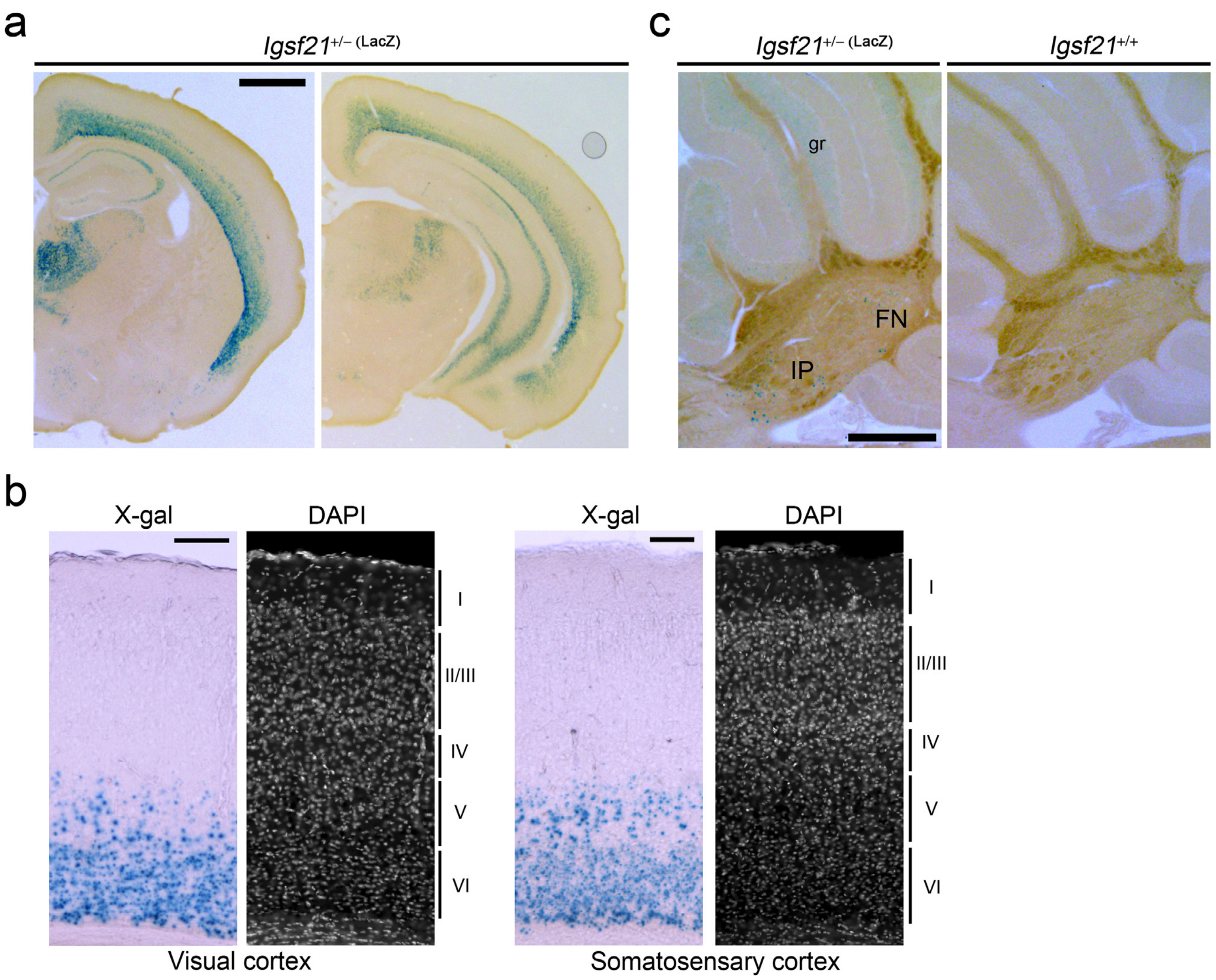

Supplementary Figure 5. *Igsf21* gene expression in the different brain regions at postnatal 4 weeks
(**a**) Images of X-gal staining of *Igsf21*$^{+/-(LacZ)}$ mouse brain coronal sections show that the *Igsf21* gene is expressed in the CA1 and CA3 regions of the dorsal (left) and ventral (right) hippocampus.
(**b**) High magnification images of X-gal staining and DAPI counterstaining of the visual cortex and the somatosensory cortex show that *Igsf21* gene expression is restricted to layers 5 (V) and 6 (VI).
(**c**) The *Igsf21* gene is expressed in a few cells in the interposed nucleus (IP) and the fastigial nucleus (FN) of the cellebellum. The *Igsf21* gene is also weakly expressed in granular layer (gr) cells. These signals are not detected in a negative control brain slice from a wild-type littermate (*Igsf21*$^{+/+}$).
Scale bars represent 1 mm (**a**), 100 μm (**b**), and 500 μm (**c**).

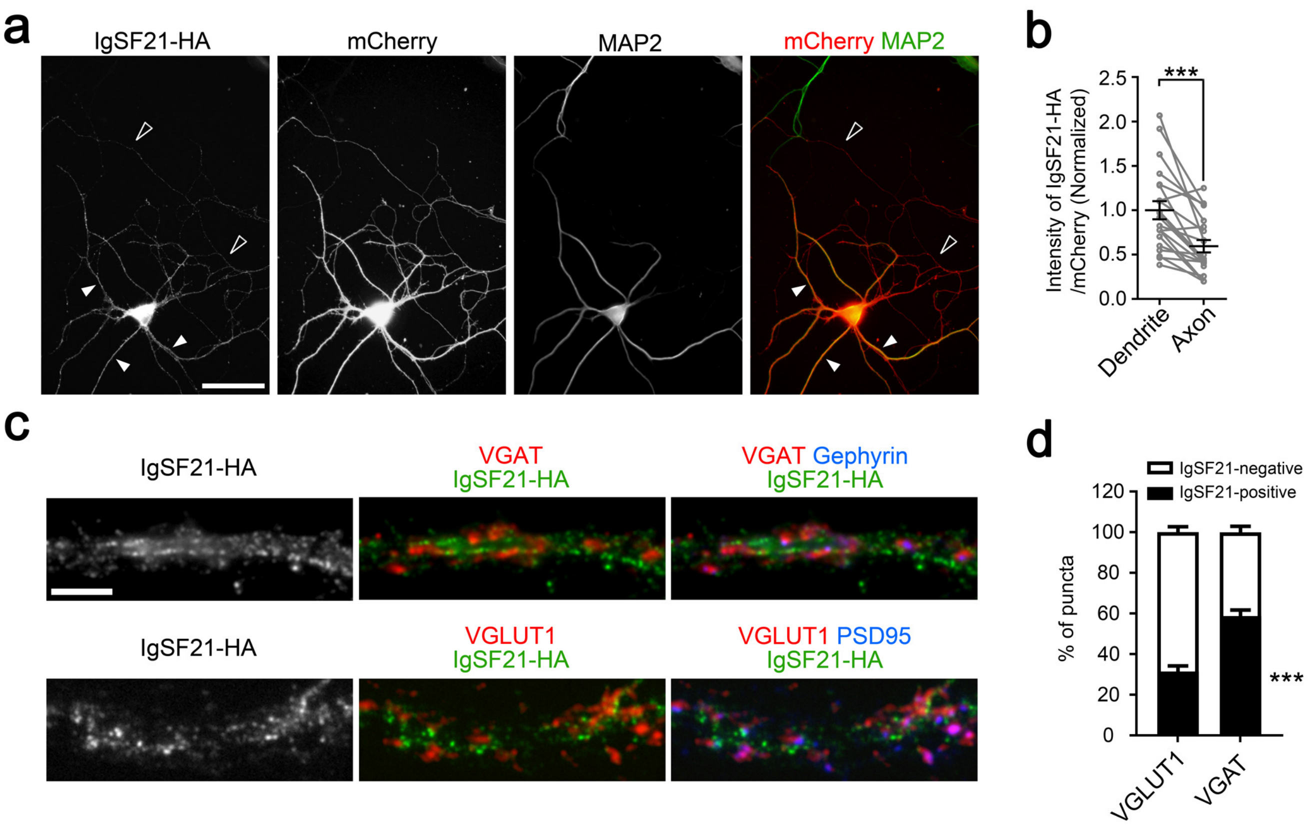

Supplementary Figure 6. IgSF21 localizes preferentially to dendrites in a synaptic punctate pattern

(**a**,**b**) IgSF21-HA expressed in hippocampal neurons together with mCherry at 14 days *in vitro* is preferentially localized at MAP2-positive dendrite sites (filled arrowheads) rather than mCherry-positive and MAP2-negative axon sites (open arrowheads). Open arrowheads indicate weak or undetectable expression of IgSF21-HA at axonal sites. n = 21 cells from three independent experiments, paired t-test, ***$P < 0.0001$. Data are presented as mean ± s.e.m.

(**c**) IgSF21-HA expressed in hippocampal neurons at 14 days *in vitro* exhibits a diffuse distribution with a punctate pattern on dendrites. Some puncta of IgSF21-HA are juxtaposed to or overlapped with a subset of VGAT puncta. Some IgSF21-HA puncta are also juxtaposed to or overlapped with a subset of VGLUT1 puncta.

(**d**) Quantification of the percentage of VGLUT1 or VGAT puncta associated with IgSF21-HA puncta. IgSF21-HA puncta are associated significantly more with VGAT puncta than with VGLUT1 puncta. n = 20 cells each from three independent experiments, two-way ANOVA, F (1, 76) = 91.7, $P < 0.0001$. ***$P < 0.0001$ for the percentage of IgSF21-positive VGAT puncta compared to that of IgSF21-positive VGLUT1 puncta by Bonferroni's multiple comparisons test.

Scale bars represent 50 μm (**a**) and 5 μm (**c**).

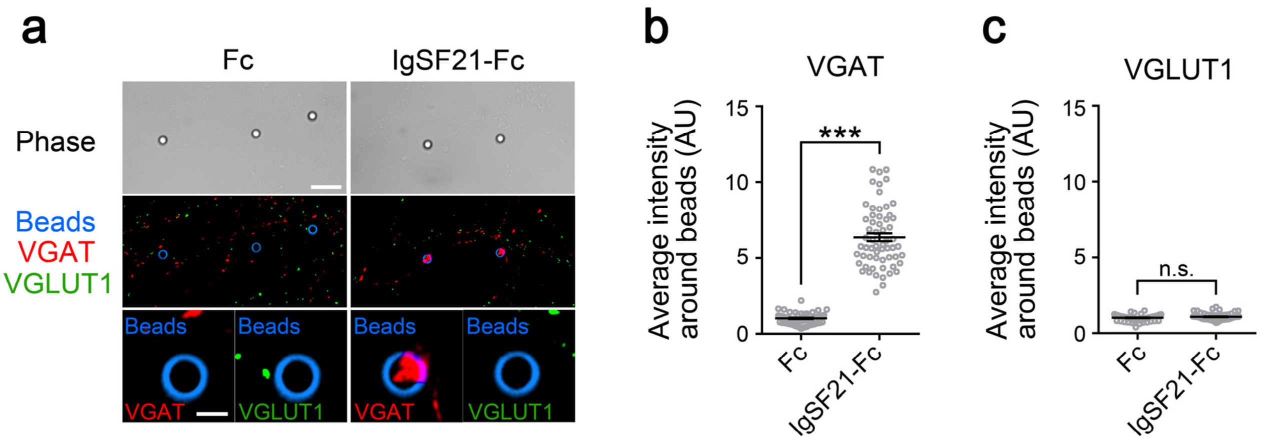

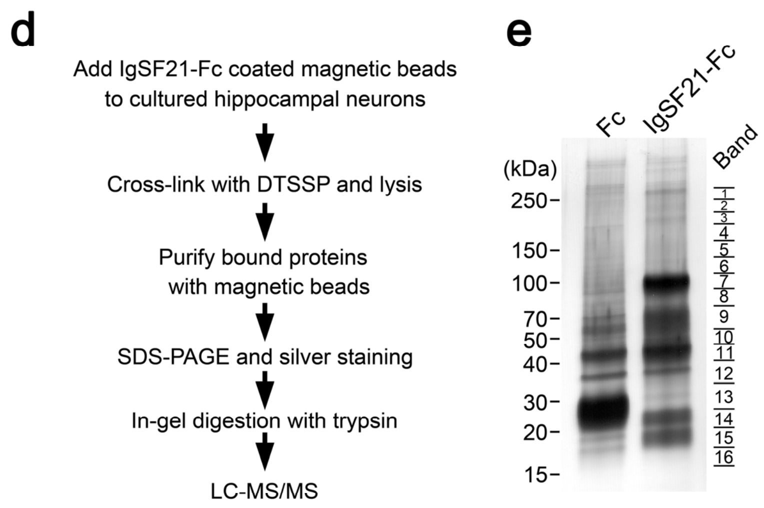

f

Band	MW (kDa)	Uniprot ID	Protein name	MASCOT Protein Score
1	~250	Q63374<br>O88778	NRX2α<br>Bassoon	438<br>104
2	150-250	Q63374<br>O88778	NRX2α<br>Bassoon	550<br>129
3	150-250	Q63374	NRX2α	289
4	150-250	Q9QXY2<br>Q63374	SRCIN1<br>NRX2α	617<br>291
5	100-150	Q63374	NRX2α	145
6	100-150	none		
7	70-100	Q64605	PTPσ	166
8-16	15-100	none		

Supplementary Figure 7. Unbiased proteomics identification of neurexin2α as an IgSF21-interacting protein

(**a**) IgSF21-Fc-coated beads (blue) induce the clustering of VGAT (red), but not VGLUT1 (green) in hippocampal neuron cultures. Scale bars represent 10 μm (top) and 2 μm (bottom).

(**b,c**) Quantification of the average intensity of VGAT (**b**) and VGLUT1 (**c**) around beads coated with IgSF21-Fc or Fc. n = 60 beads each from two independent experiments, Mann-Whitney test, ***P < 0.0001 for VGAT and P = 0.2703 for VGLUT1, compared with Fc. n.s., not significant. Data are presented as mean ± s.e.m.

(**d-f**) Schema for isolating presynaptic proteins interacting with IgSF21 (**d**). Proteomic screen for IgSF21-interacting proteins using IgSF21-Fc-coated magnetic beads plated onto hippocampal neuron cultures. After protein cross-linking and solubilization, the protein samples magnetically-isolated by IgSF21-Fc- and Fc-coated beads were subjected to SDS-PAGE followed by silver staining (**e**). 16 bands were cut from each lane. Mass spectrometry analysis (**f**) identified several proteins with a Mascot protein score of over 100 that were present only in the IgSF21-Fc fraction.

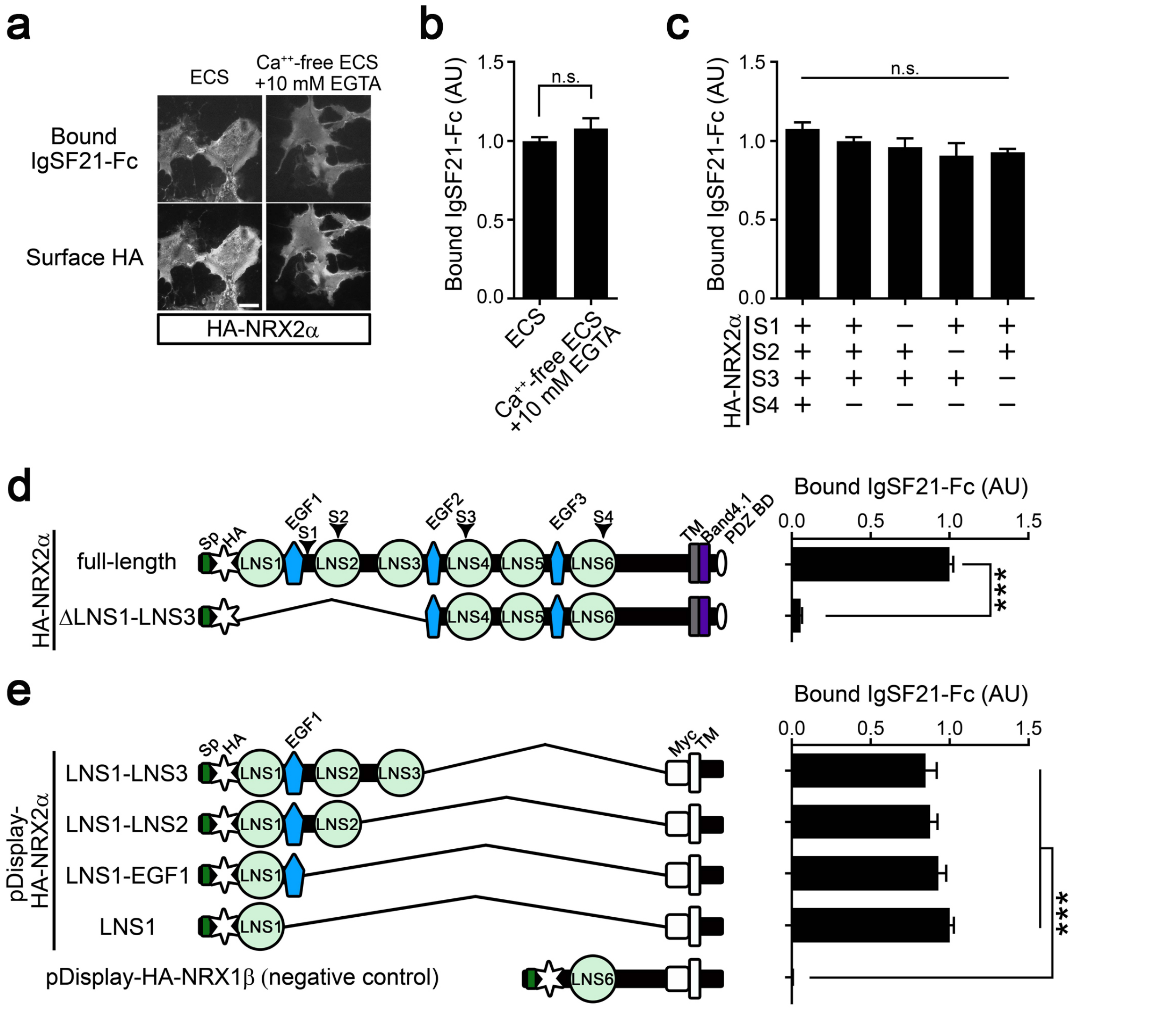

Supplementary Figure 8. Ca^{++}-independent and neurexin2α splicing-independent interaction of IgSF21 with neurexin2α and domain analysis of the interaction

(**a,b**) IgSF21-Fc protein binds HA-tagged neurexin2α (HA-NRX2α) independently of extracellular Ca^{++}. n = 30 cells each from three independent experiments, Mann-Whitney test, P = 0.5229. n.s., not significant. ECS, extracellular solution. A scale bar represents 30 μm.

(**c**) IgSF21-Fc protein binds HA-NRX2α independently of NRX2α splicing at sites S1-S4. n = 30 cells each from three independent experiments, one-way ANOVA, P = 0.1195. n.s., not significant.

(**d**) IgSF21-Fc proteins do not bind an HA-NRX2α mutant lacking the first four domains (LNS1, EGF1, LNS2, and LNS3). n = 30 cells each from three independent experiments, unpaired t-test, ***P < 0.0001.

(**e**) IgSF21-Fc proteins bind all of the pDisplay-based HA-NRX2α constructs including the one that retains only LNS1. IgSF21-Fc does not bind a negative control, the pDisplay-based HA-NRX1β construct. n = 30 cells each from three independent experiments, Kruskal-Wallis one-way ANOVA, P < 0.0001. ***P < 0.0001 compared with HA-NRX1β by Dunn's multiple comparisons test.

Sp, signal peptide; HA, hemagglutinin tag; EGF, epidermal growth factor domain; LNS, laminin/neurexin/sex hormone-binding globulin-domain; Myc, myc tag; TM, transmembrane region; PDZ BD, PDZ-binding domain.

Data are presented as mean ± s.e.m.

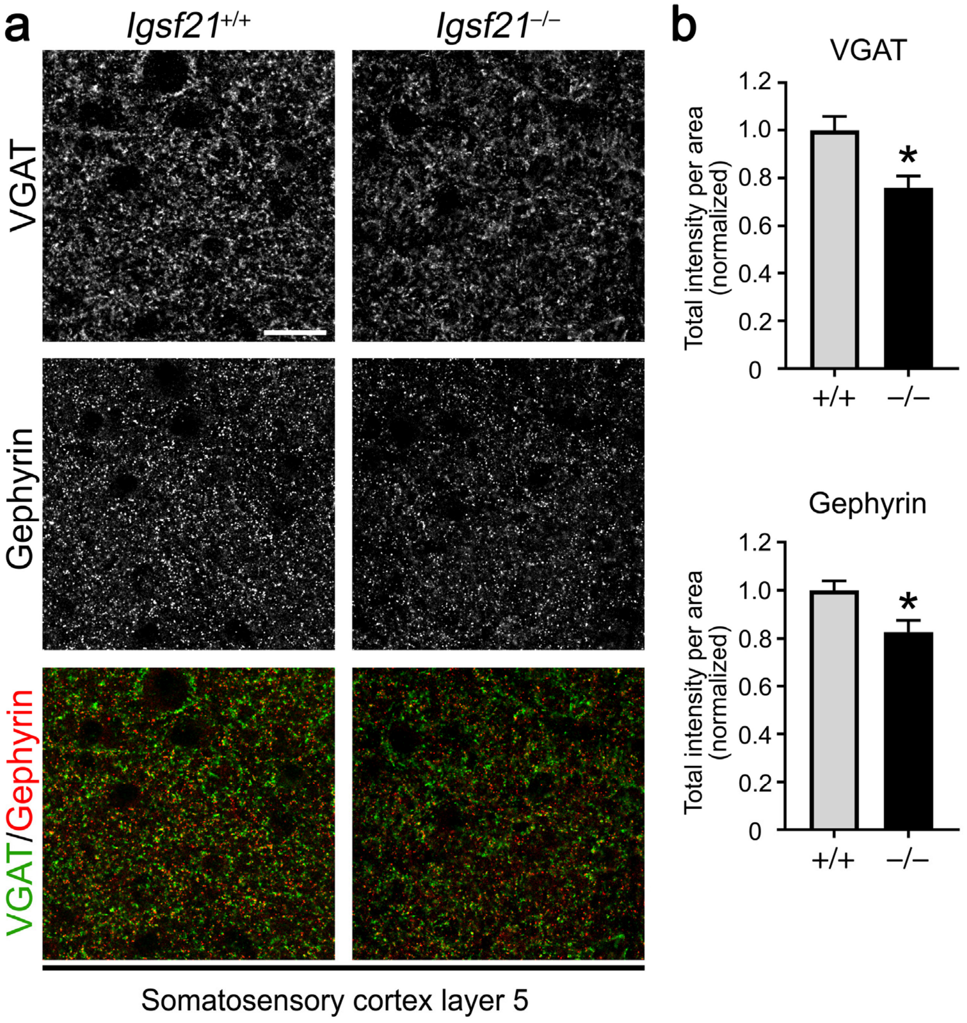

Supplementary Figure 9. VGAT and gephyrin immunofluorescence is reduced in layer 5 of the somatosensory cortex in *Igsf21*$^{-/-}$ mice

(**a**) Double immunostaining for VGAT and gephyrin in layer 5 of the somatosensory cortex of *Igsf21*$^{+/+}$ and *Igsf21*$^{-/-}$ mice. The VGAT and gephyrin intensity is significantly decreased in *Igsf21*$^{-/-}$ mice when compared to that in *Igsf21*$^{+/+}$ littermates.

(**b**) Quantification of the total intensity of VGAT and gephyrin puncta in layer 5 of the somatosensory cortex. VGAT and gephyrin intensity was measured in the same fields. Intensity values are normalized to the mean value of *Igsf21*$^{+/+}$ control. n = 36 fields from 4 *Igsf21*$^{+/+}$ mice and 4 *Igsf21*$^{-/-}$ mice. Mann-Whitney test, **P* = 0.003 (VGAT), **P* = 0.0023 (gephyrin).

Scale bar represents 20 μm. Data are presented as mean ± s.e.m.

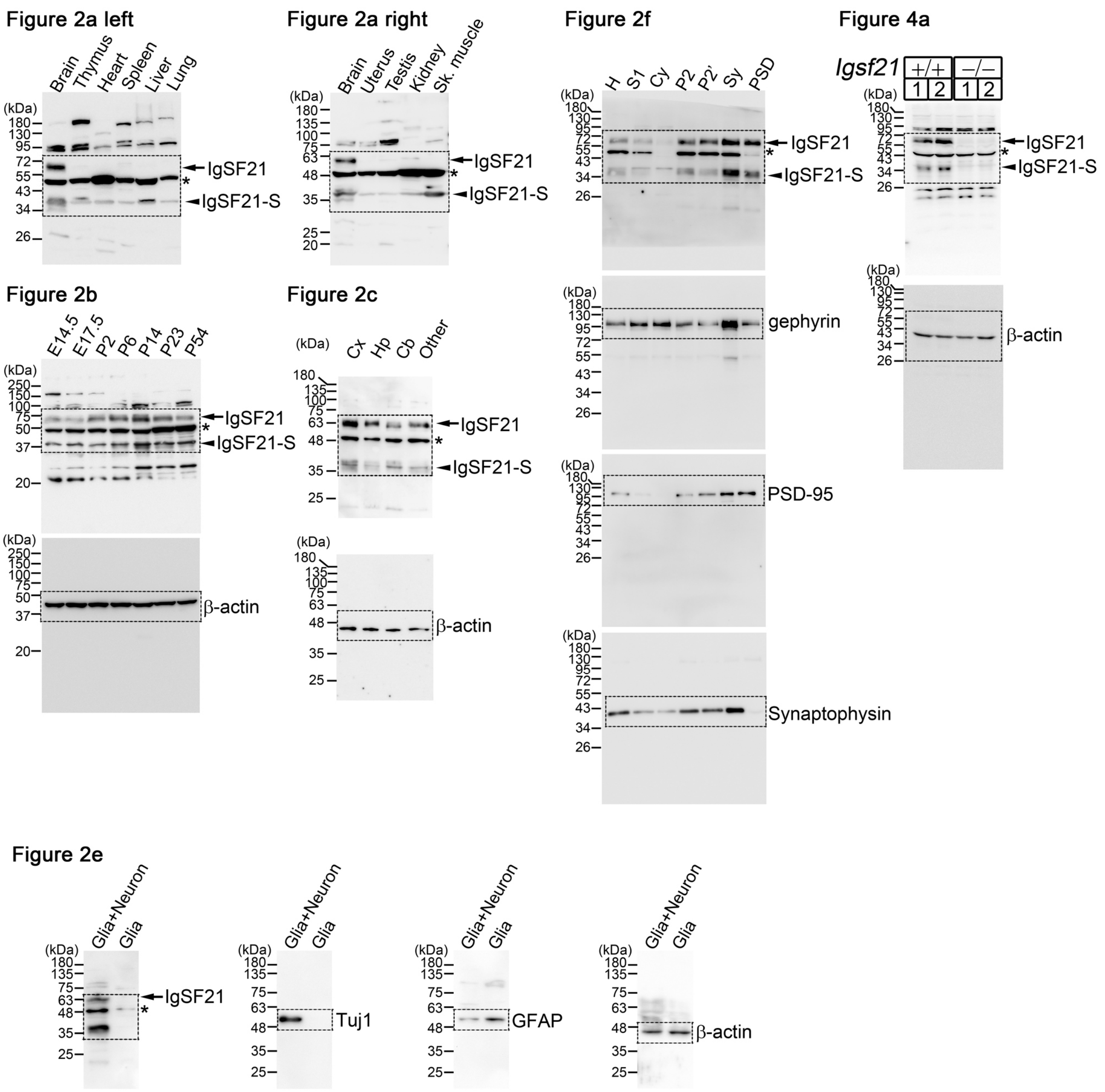

Supplementary Figure 10. Full-length images of immunoblots
Figure 2a, 2b, 2c, 2e, 2f and Figure 4a

Figure 4c, Cortex

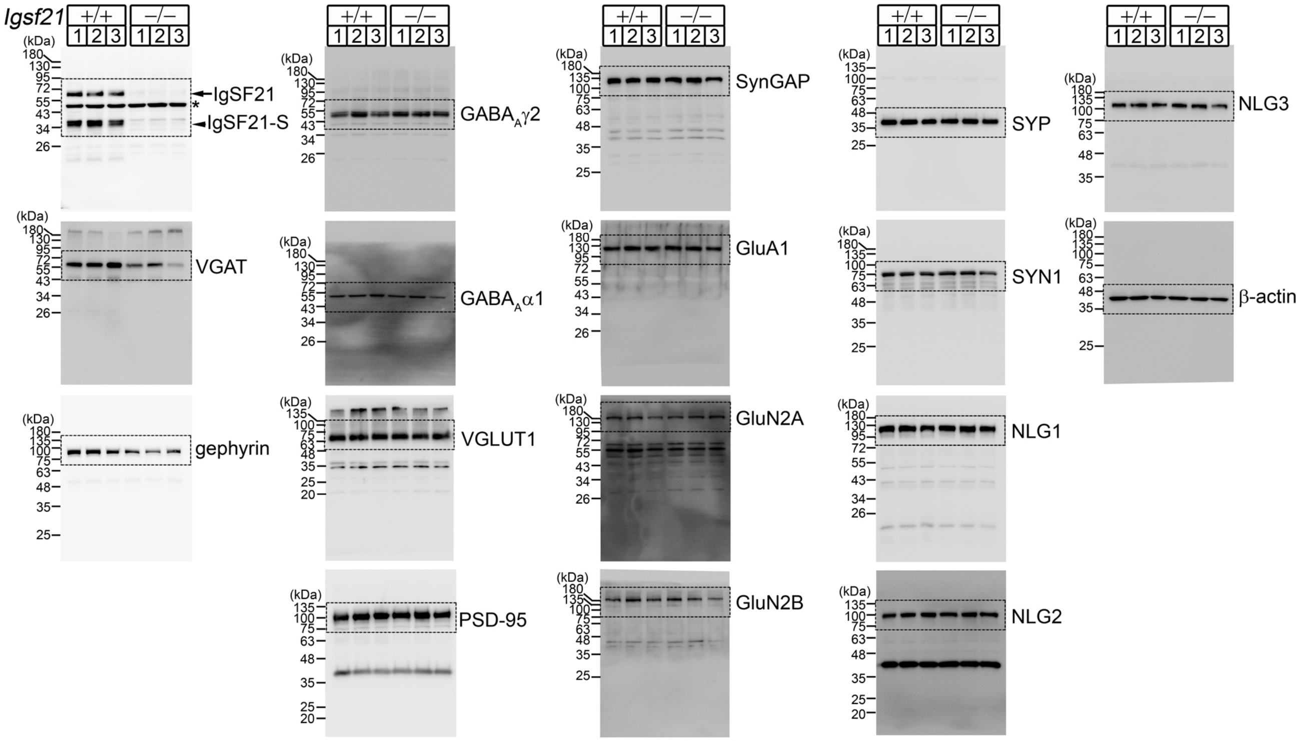

Figure 4c, Hippocampus

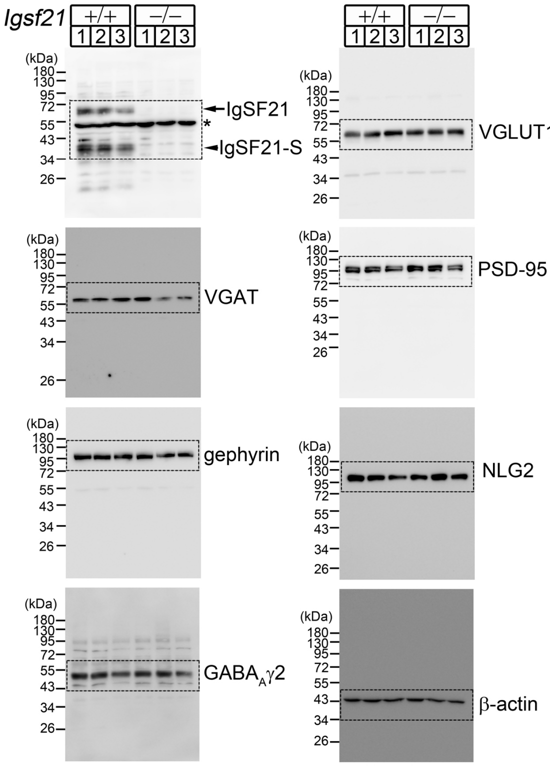

Supplementary Figure 1f

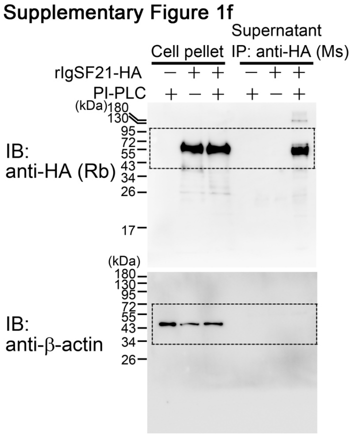

Supplementary Figure 11. Full-length images of immunoblots

Figure 4c and Supplementary Figure 1f
